# Two Auxinic Herbicides Affect *Brassica napus* Plant Hormone Levels and Induce Molecular Changes in Transcription

**DOI:** 10.3390/biom11081153

**Published:** 2021-08-04

**Authors:** Jutta Ludwig-Müller, Roman Rattunde, Sabine Rößler, Katja Liedel, Freia Benade, Agnes Rost, Jörg Becker

**Affiliations:** 1 Institut für Botanik, Technische Universität Dresden, 01062 Dresden, Germany; r-rattunde@online.de (R.R.); sabine.roessler@tu-dresden.de (S.R.); Katja.Liedel@gmx.de (K.L.); freia.benade@tu-dresden.de (F.B.); 2 Corteva Agriscience Germany GmbH, Riedenburger Str. 7, 81677 München, Germany; agnes.rost@corteva.com (A.R.); joerg.becker@corteva.com (J.B.)

**Keywords:** abscisic acid, aminocyclopropanecarboxylic acid, aminopyralid, auxinic herbicides, *Brassica napus*, ethylene, halauxifen-methyl, *GH3* genes, indole-3-acetic acid, picloram

## Abstract

With the introduction of the new auxinic herbicide halauxifen-methyl into the oilseed rape (*Brassica napus*) market, there is a need to understand how this new molecule interacts with indigenous plant hormones (e.g., IAA) in terms of crop response. The aim of this study was to investigate the molecular background by using different growth conditions under which three different auxinic herbicides were administered. These were halauxifen-methyl (Hal), alone and together with aminopyralid (AP) as well as picloram (Pic). Three different hormone classes were determined, free and conjugated indole-3-acetic acid (IAA), aminocyclopropane carboxylic acid (ACC) as a precursor for ethylene, and abscisic acid (ABA) at two different temperatures and growth stages as well as over time (2–168 h after treatment). At 15 °C growth temperature, the effect was more pronounced than at 9 °C, and generally, the younger leaves independent of the developmental stage showed a larger effect on the alterations of hormones. IAA and ACC showed reproducible alterations after auxinic herbicide treatments over time, while ABA did not. Finally, a transcriptome analysis after treatment with two auxinic herbicides, Hal and Pic, showed different expression patterns. Hal treatment leads to the upregulation of auxin and hormone responses at 48 h and 96 h. Pic treatment induced the hormone/auxin response already after 2 h, and this continued for the other time points. The more detailed analysis of the auxin response in the datasets indicate a role for *GH3* genes and genes encoding auxin efflux proteins. The upregulation of the *GH3* genes correlates with the increase in conjugated IAA at the same time points and treatments. Also, genes for were found that confirm the upregulation of the ethylene pathway.

## 1. Introduction

Plant hormones can be sorted into different classes, depending on their chemical structures. Research into plant hormones initially identified five major classes: abscisic acid, auxin, cytokinins, ethylene, and gibberellins. This list was later expanded, and brassinosteroids, jasmonates, salicylic acid, and strigolactones are now considered as major plant hormones as well [1]. Their interaction to control the specific growth and developmental processes is long known. Among these, auxins have been known from early on to display a concentration-dependent effect different from that of other hormone classes. While the majority of plant hormones show a concentration-dependent saturation curve, auxins follow in many cases an optimum curve for growth [2]. Therefore, the proper control of tissue-specific concentrations is essential for growth and development [3]. 

**Figure 1:**
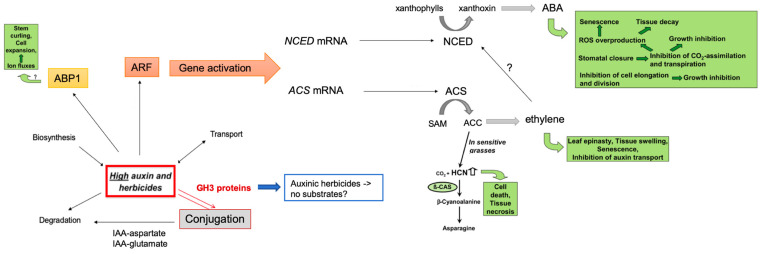
Interplay between IAA, auxinic herbicides, ethylene, and ABA. Adapted from [3,5]. The scheme on the regulation of free IAA by different possibilities takes specifically the synthesis of conjugates by GH3 amino acid conjugate synthetases into account. High auxin could induce gene expression of ethylene and ABA synthesis genes, leading to the events in the green boxes, which ultimately damage the plant.

Synthetic auxins were developed in the late 1940s because of their selective action to preferentially control dicot weeds, are in selective herbicides for cereal crops, and belong to the most important herbicides to control weed growth in agriculture worldwide. Following this, the development of several auxinic herbicides has been reported. Natural auxins are usually inactivated very quickly by conjugation and degradation, while auxinic herbicides are retained for longer periods of time [4]. Hormone interplay is not only important in the regulation of plant growth and development but also for the growth-inhibiting effect of auxinic herbicides. There are several possible mechanisms involved in high auxins to control plant death mediated by multiple following hormones such as ethylene and abscisic acid (Figure 1) [5,6].

The interplay between high auxin (or auxinic herbicides) and ethylene as well as ABA has been described [5]. The model in Figure 1 proposes another pathway, namely auxin conjugation, to be important. Several publications have indicated that the auxinic herbicides might not be conjugated to the inactive amino acid conjugates, thereby interfering with the control of auxin levels [7]. For *Arabidopsis thaliana*, it was shown by microarray and qPCR analysis that one *GH3* gene (*GH3.3*) was strongly upregulated after treatment with the auxinic herbicide dicamba [4]. While IAA is conjugated to an amino acid and thereby the pool of free IAA reduced [3], auxinic herbicides are thought not to be a substrate for GH3 proteins, even though they were able to upregulate gene expression [7]. Furthermore, in this work, an upregulation of a marker gene for ABA (*NCED3*) and ethylene (*ACS11*) biosynthesis was upregulated on the transcript level [4]. However, there is one report on the conjugation of dicamba to glutamate by some plant enzymes [8], but these are only in vitro data, and the situation in planta might be different. In addition, a *Pisum sativum* (pea) auxin conjugate synthetase was upregulated by IAA, 2,4-dichloroacetic acid (2,4-D) and the two auxinic herbicides dicamba and picloram (Pic) on the gene expression and enzymatic level [9]. For other major crop plants, such as *Brassica napus* (oilseed rape), such investigations have not been performed as yet.

**Figure 2:**
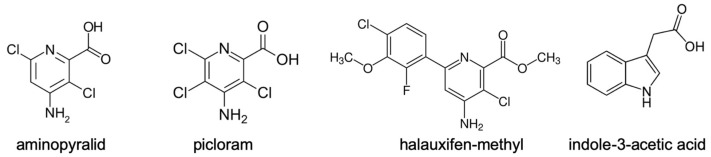
Structures of auxinic herbicides used in this study in comparison to IAA. Structures from Wikimedia [17,18,19,20].

Auxinic herbicide formulations have been described for different compound classes, such as 2,4-D [7] and later dicamba or Pic [4,5,10,11], and their mode of action has been investigated. The development of novel auxinic herbicides with a better effect on dicots has been described [12]. Among these are also combinations of different auxinic compounds. Halauxifen-methyl (Hal) is contained in the new oilseed rape herbicide Belkar™ [12], which combines the two active ingredients, Hal and Pic in a premix formulation (see Figure 2 for all structures in comparison to IAA). Since specifically halauxifen-methyl is still not so often used, it can be regarded as a good alternative in broadleaf weed control [13]. 

Auxinic herbicides can cause typical symptoms sometimes also in *B. napus* plants under field conditions such as epinastic movement or lightening and changes in leaf veins. Therefore, it was decided to investigate the hormonal basis of this phenotype in situations, when halauxifen-methyl had been applied alone or in tank-mix together with aminopyralid (AP), which is another synthetic auxin. The following questions were addressed: (1) What is the impact of Hal, the combination of Hal + AP as well as Pic alone on the plant hormones IAA and abscisic acid when compared to untreated control? (2) How does temperature (e.g., 9 °C vs. 15 °C) influence the impact of synthetic hormones on IAA and abscisic acid level? (3) How do the development stage or leaf age of oilseed rape influence the impact of synthetic hormones on IAA and abscisic acid level? (4) During which time frame after treatment are the possible alterations in hormones visible? Based on the data available on the interaction of auxinic herbicides with other plant hormones [5], first free IAA and as a stress hormone ABA were measured. Since no reproducible effect for ABA was found, ACC as the precursor of ethylene was included in a second experimental set. Based on the evidences from the literature that auxin conjugation might be an important factor for the effect of auxinic herbicides [4,8,9], the levels of conjugated IAA in addition to free IAA were also determined. 

Finally, to get a better overview on the transcriptome level, three time points for two different treatments were compared for Hal and Pic since so far, in the literature, only transcriptome data for 2,4-D and dicamba are available in the model organism *A. thaliana* [4,6,14]. Very recently, the short time effect of halauxifen-methyl was investigated on the transcriptome of a weed species, *Erigeron canadensis*, that can be controlled in the field by Hal [15,16]. While in the latter study, a target weed species has been used and treatment with 2,4-D, dicamba, and Hal were investigated, in our work, we investigated the transcriptome of a crop species after Hal and Pic treatments. The differentially expressed genes in our work were monitored with an emphasis on auxin-related genes, especially *GH3* genes. Comparisons to [16] show interesting differences between a crop and weed species. 

## 2. Materials and Methods

### 2.1. Plant Growth and Treatment

The growth conditions chosen should mimic the conditions in the field at the time of herbicide application in late summer/autumn. *Brassica napus* (cv. Visby, obtained from Sächsisches Landesamt für Umwelt, Landwirtschaft und Geologie, Nossen, Germany) seeds were germinated on a wet paper towel for 3–5 days at 15 °C and an approximate humidity of 60% until most of the hypocotyls showed both cotyledons. Then, the seedlings were transferred to soil with four plants per pot and five pots per tray and were incubated under the selected temperature in climate chambers with 12 h light exposure to the growth stage BBCH11 or BBCH14 (BBCH stands for Biologische Bundesanstalt, Bundessortenamt und Chemische Industrie) [21,22]. In the first experiment for BBCH14, all leaves were collected together; in the second experiment, leaves 1 and 2 as well as 3 and 4 were harvested as mixed samples, and the sample G (newly grown part) was harvested separately. To identify the influence of the temperature on the effect of Hal, the plants were grown after their initial period at 15 °C further at 15 °C or at 9 °C. The temperature did not vary between day and night. 

To identify the suitable herbicide concentrations, the following concentrations were applied to stage BBCH11: 0.5 µg/plant and 1.0 µg/plant Hal. The concentrations were chosen according to the recommended concentration for field treatments. For subsequent experiments, the plants were treated with auxinic herbicides as follows (Table S1): halauxifen-methyl tech: 0.5 or 1.0 µg active ingredient (ai)/plant; aminopyralid: 1.8 µg ai/plant; picloram tech: 2.4 µg ai/plant. Controls were treated with the solvent only.

Working solutions for herbicide application were prepared as the following (Table 1): Hal was dissolved in DMSO to obtain a 100× stock solution (10 g/L), and the same was applied for AP (36 g/L). Then, the Hal stock solution was incubated with 1M NaOH in a 1:1 ratio for 5 min at room temperature. After that, 0.1% Triton X-100 was added 1:10, and the solution was adjusted to the working concentration (1×) with distilled H_2_O. Application was performed with an automatic pipette. One drop consisted of 1 µL substance (stock solution 0.1 µg/µL) for BBCH11 and 2.5 µL for BBCH14 (see Figure S1 for the application points).

Plants were harvested at the following time points: 2 h (day of treatment), 4 h (day of treatment), 24 h (1 day), 48 h (2 days), 72 h (3 days), 96 h (4 days), 120 h (5 days) –168 h (7 days). The leaf tissue was frozen in liquid nitrogen and kept until analysis. The plant materials for the temperature dependence, the developmental experiment, and the time course were grown at different time points, the plants for the determination of free and total IAA and for RNAseq were from the same batch. The leaves were harvested; then, their fresh weight was determined, and they were frozen in liquid nitrogen and stored until analysis at –80 °C. The respective incubation times are given in the figures and respective legends and in the respective experiments in more detail, since not all time points were used for all treatments.

### 2.2. Determination of IAA, ABA, and ACC 

#### 2.2.1. Source of Heavy Isotope Labeled Standards

To all samples, heavy isotope labeled standards were added, allowing quantification. ^13^C_6_-IAA and d_6_-ABA were obtained from Cambridge Isotope Laboratories (Andover, MA, USA), d_4_-ACC from Euriso-Top GmbH (Saarbrücken, Germany). Then, the ratios between masses (endogenous and standard) are used to calculate the concentration of a compound of interest. The characteristic ions for IAA were 130/136 (endogenous and heavy labelled), for ACC 141/145 and for ABA 190/194 (note that for ABA, two deuterium are lost during fragmentation in the MS).

#### 2.2.2. Free Acidic Hormones

The hormone determination protocol changed during the course of the experiments since it was desired to extract not only IAA and ABA but also ACC from the leaves. Therefore, for the first set of experiments, the following method was used [23] that allowed the simultaneous determination of IAA and ABA. Sample values were always biological triplicates of plants grown at the same period with 3 technical replicates for one data point. Additional individual data points in the time course were from additional experiments performed at different years.

(I) The leaves of mixed samples were homogenized in 3 mL of extraction buffer (isopropanol + 1% acetic acid) with sea sand in a mortar at room temperature after adding the following heavy isotope labeled standards: 300 ng ^13^C_6_-IAA and 600 ng d_6_-ABA (100 and 200 ng/technical replicate, respectively). Then, the homogenate was split in three reaction tubes, each representing one technical replicate, and was centrifuged at 13,000 rpm and 8 °C for 10 min. The liquid supernatant was transferred to a fresh reaction tube together with 150 μL distilled H_2_O and mixed by inverting. The organic phase was reduced under constant N_2_ flow at room temperature. The pH of the aqueous phase was adjusted to pH 3. The sample was extracted twice by adding 500 μL of ethyl acetate and centrifugation at 13,000 rpm and 8 °C. The solvent phases were combined in a glass vial and were dried completely under constant N_2_ flow at room temperature. The solid contents were dissolved in 200 μL of MeOH and were incubated with 200 μL of Diazo-reagent (1:100 (trimethylsilyl)-diazomethane in diethylether, Sigma-Aldrich, München, Germany) for 30 min at room temperature to obtain methylated hormones for gas chromatographic analysis [24]. The solvent was completely dried under constant N_2_ flow at room temperature. The solid remains were dissolved in 30 μL ethyl acetate of which 1 μL was injected.

(II) To determine acidic plant hormones including ACC in one extraction and analysis, a method was adapted using the protocols of [25] and [26] with an additional methylation of the samples using trimethylsilyldiazomethane [24]. The frozen leaf tissues (300 mg) were first ground to a fine powder in the presence of liquid nitrogen by mortar and pestle and transferred to a 2 mL microcentrifuge tube. To the ground tissue, heavy isotope labeled standards were added at final concentrations of IAA (100 ng), ACC (100 ng), and ABA (200 ng) per technical replicate. 

The sample was suspended in 200 μL of a sodium hydroxide (1% *w*/*v*) solution, and 147 μL of methanol and 34 μL of pyridine were added to the solution. Then, the samples were mixed vigorously by vortex for 25–30 s. Then, methyl chloroformate (20 μL) was added, and the suspension was again vigorously mixed for 25–30 s. A second volume of methyl chloroformate (20 μL) was added, and the samples were again mixed for 25–30 s. Subsequently, chloroform (400 μL) was added, the sample was mixed for 10 s, and a 50 mM sodium bicarbonate solution (400 μL) was added. Following further mixing for 10–15 s, the extract was separated into two phases by centrifugation for 30 s at 16,000× *g* and room temperature. The lower organic layer containing the phytohormones was transferred by pipette to a fresh microcentrifuge tube, being careful not to disturb the layer of plant debris separating the aqueous and organic layers. Then, anhydrous sodium sulfate was added until the sodium sulfate crystallized. The sample was centrifuged for 30 s at 16,000× *g* to pellet the sodium sulfate at room temperature. The liquid phase was transferred to a glass vial and dried completely under N_2_. For methylation, 200 μL of methanol and 200 μL of diazo-reagent were added to the dried samples and incubated for 30 min at room temperature. The solvent was completely dried under N_2_ and the sample was dissolved in 50 μL ethyl acetate, of which 1 μL was analyzed by GC-MS (see Section 2.2.4).

#### 2.2.3. Total IAA

For total IAA, the samples were hydrolyzed with a final concentration of 7 M NaOH [27]. The hydrolysis step was incorporated in the newly developed method described above. As a confirmation of hydrolysis, one sample with an IAA-Asp was used, which resulted in the presence of IAA. In addition, it was tested by adding free IAA that there was no high IAA degradation (Figure S2). After the addition of 200 μL of labeled standard H_2_O, we added 200 μL of 14 M sodium hydroxide and mixed thoroughly. The sample was incubated for 3 h under constant N_2_ flow at 90 °C. Then, the sample temperature was cooled down on ice. Debris that was formed during the incubation was pelleted by centrifugation for 5 min at 16,000× rpm, and the supernatant was transferred to a fresh tube. Then, the pH was adjusted to pH 7 by adding 80 μL of HCl (37%) and proceeded with 2 M HCl in 20 μL steps where the pH was checked after every addition with indicator paper. After the pH was adjusted, the samples were further extracted as described above by the addition of 1% sodium hydroxide solution. To test the efficiency of the hydrolysis, unlabeled IAA and IAA-aspartate were added and analyzed after alkaline hydrolysis after the protocol described (Figure S2).

#### 2.2.4. GC-MS Analysis

Gas chromatography-mass spectrometry (GC-MS) analysis was carried out on a Varian Saturn 2100 ion-trap mass spectrometer using electron impact ionization at 70 eV, connected to a Varian CP-3900 gas chromatograph equipped with a CP-8400 autosampler (Varian, Walnut Creek, CA, USA). For the analysis, 1 µL of the methylated sample dissolved in ethyl acetate was injected in the splitless mode (splitter opening 1:100 after 1 min) onto a Phenomenex ZB-5 column, 30 m × 0.25 mm × 0.25 µm using He carrier gas at 1 mL min^−1^. The injector temperature was 250 °C and the temperature program was 70 °C for 1 min, followed by an increase of 20 °C min^−1^ to 280 °C, then 5 min isothermically at 280 °C. The transfer line temperature was 280 °C. The scan rate was 0.6 s scan^−1^, the multiplier offset voltage was 200 V, the emission current was 30 µA, and the trap temperature was 200 °C. IAA, ABA, and ACC were identified according to the retention time on GC compared with an authentic methylated standard and by recording the respective mass spectrum. The analytics was adapted for measuring all components in one run (IAA, ABA, ACC). All compounds were separated from each other (Figure S2). Finally, the data were calculated using the isotope dilution equation as described in [28]. The method relies on the addition of standards that are labeled with heavy isotopes. The peak area of a mass was recorded, and from that, the calculations were done. The characteristic ions for IAA were 130/136 (endogenous and heavy labeled), for ACC 141/145, and for ABA 190/194. Note that for ABA, two deuterium are lost during fragmentation in the MS.

### 2.3. Transcriptome Analysis

#### 2.3.1. RNA Extraction

RNA extraction was performed with NucleoZOL (Macherey & Nagel, Düren, Germany) according to the manufacturer’s instructions. Samples were homogenized with mortar and pestle under constant supply of liquid nitrogen to a fine powder. Per 50 mg of tissue, 500 μL NucleoZOL were pipetted to the ground sample in the mortar and distributed with the pestle, covering the tissue powder completely (NucleoZOL might freeze, city, State abbreviation and country). After thawing, the complete mix was pipetted into a plastic tube using a cut tip. To precipitate contaminants, 200 μL of RNase-free water per 500 μL of NucleoZOL lysate was added. Then, the sample was vigorously shaken for 15 s and incubated at room temperature for 15 min. Then, the sample was centrifuged for 15 min at 12,000× *g* at room temperature, and 500 μL of the supernatant was transferred to a fresh tube. To precipitate the total RNA, 1 volume of isopropanol to 1 volume supernatant was added, and the sample was incubated at room temperature for 10 min, which was followed by centrifugation for 10 min at 12,000× *g*. The supernatant was discarded, and the pellet containing the RNA was washed in 500 μL 75% ethanol followed by centrifugation for 3 min at 8000× *g*. The ethanol was removed from the pellet using a pipette and the washing step was repeated. Afterwards, the ethanol was evaporated as completely as possible by drying in air. Then, the RNA pellet was dissolved in RNase-free water to obtain an RNA concentration of 1–2 g/μL, and RNAse inhibitor was added to the sample. Isolated RNA was quantified with an ND-1000 Spectrophotometer (NanoDrop Technologies, Inc., Wilmington, DE, USA), and the integrity of the RNA was checked with the Agilent 2100 Bioanalyzer platform using the RNA 6000 Pico Kit (Agilent Technologies Manufacturing GmbH & Co. KG, Waldbronn, Germany) prior to shipping.

#### 2.3.2. RNAseq

The experimental details were taken from the data provided by Novogene (UK Sequencing Center, Cambridge, UK). Further quality controls were done by the company Novogene, where the RNAseq was performed. All samples had a high quality as confirmed. The workflow is shown in Figure S3. From the total RNA, the library was constructed; then, RNA sequencing was carried out via Illumina (San Diego, CA, USA) platforms, based on the mechanism of SBS (sequencing by synthesis), and followed by bioinformatics analysis. The following treatments were analyzed and compared to each other: control treatment 2, 48, and 96 h; Hal treatment and Pic treatment at the same time points. The detailed method used and supplied by Novogene is given in the supplement (Supplementary method). 

Gene expression for *GH3* genes was calculated manually using directly the excel files provided that included the individual reads for each sample and transcript. The gene numbers and predictions for auxin conjugation were from [29]. The gene numbers were used as a search term, the numbers for the three replicates were taken as mean, and a ratio for control and treatment with auxinic herbicide was calculated. This was the basis for the color code in the heatmap (see Section 3.5).

## 3. Results

### 3.1. Identification of the Hormonal Basis of Different Auxinic Herbicide Treatments on B. napus

**Figure 3:**
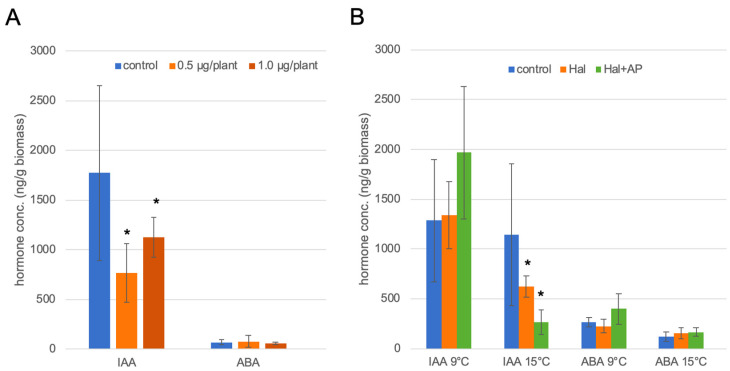
(**A**) Effect of two Hal concentrations on IAA and ABA after 96 h and at 15 °C growth temperature. (**B**) Temperature effect of Hal and AP treatment at the lower concentration on IAA and ABA after 96 h. The data are mean values of three technical replicates from one experiment. The data are all from experiments carried out at different time periods. The asterisks denote significant differences *p* < 0.05. The growth conditions chosen should mimic the conditions in the field at the time of herbicide application in late summer/autumn.

**Figure 4:**
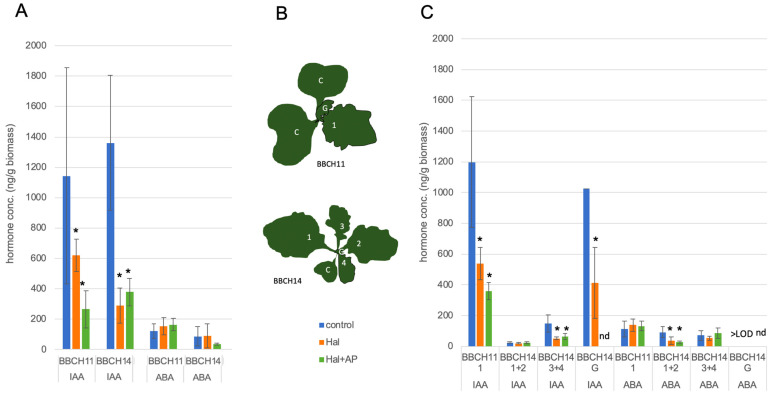
The effect of Hal (and AP) on IAA levels for different *B. napus* growth stages, BBCH11 and BBCH14, incubation at 15 °C for 96 h, AP at 1.8 µg/plant. Hal at 0.5 µg/plant for stage BBCH11; 1.5 µg/plant for stage BBCH14; the application points are shown in Figure S1. The application was performed accordingly on different leaves for BBCH14 in contrast to the younger seedlings. The experiment was performed at 15 °C, and incubation time was 96 h. (**A**) All leaves were harvested together. (**B)** The individual leaves for panel C (C = cotyledons, G = growing middle part; 1–4 following leaves). (**C**) The same experiment, but the different leaves were harvested separately for the analysis. LOD = level of detection; nd = not determined. The asterisks denote significant differences *p* < 0.05.

**Figure 5:**
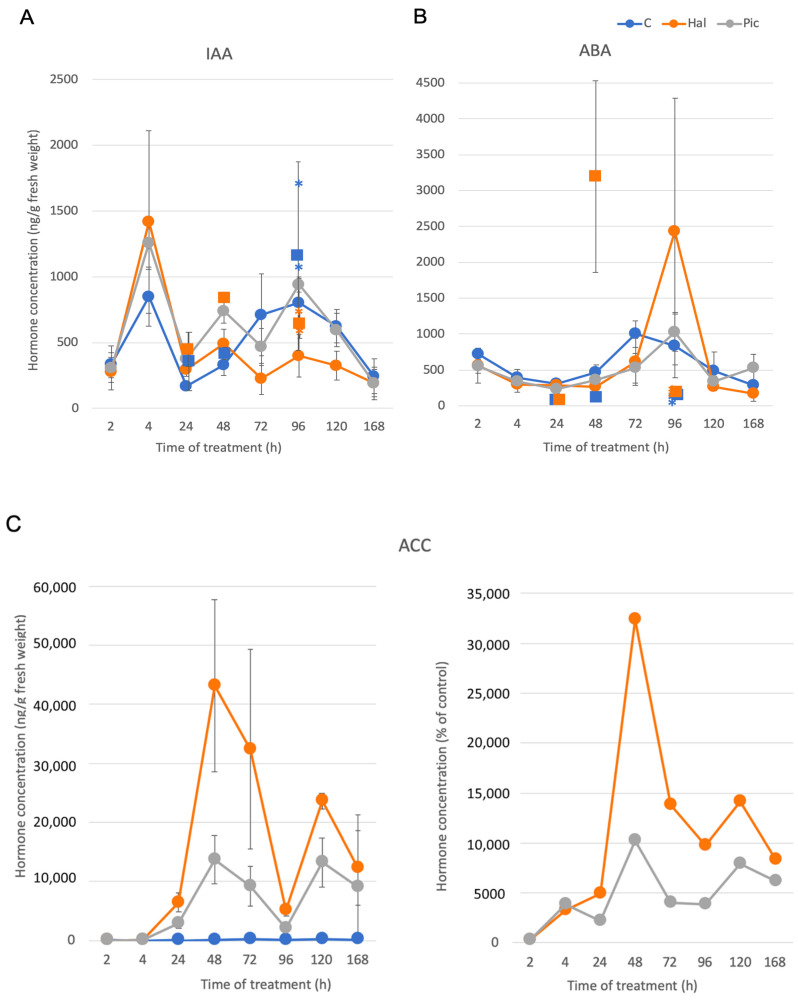
Time course of IAA (**A**), ABA (**B**), and ACC (**C**) between 2 and 168 h after treating plants with control solution and solutions containing Hal and Pic. Additional individual data points for Hal treatment (squares) in the time course were from additional experiments performed at different years. Please note the differences in the scale of the y-axis. Data are means of three biological and two to three technical experiments. The squares + SE are from a separate experimental set of plants grown at a different time point and not shown in any other figure; the blue and orange asterisks indicate the values for that time point shown in Figure 3A,B. The second panel in Figure 5C indicates the percentage of ACC after treatment compared to controls.

The aim was to measure IAA as the major auxin and ABA as a stress hormone during the treatment of plant species *B. napus*, which is not a target of auxinic herbicides in the field, with different halauxifen-methyl (Hal) concentrations over several incubation times and growth at different temperature (15 °C vs. 9 °C) as well as two different growth stages BBCH11 vs. BBCH14 (Figure 3, Figure 4 and Figure 5). In addition, the combinatorial effect with a second herbicide (aminopyralid, AP) was investigated, where the latter was not altering the results, only in some cases did it increase the effect seen on IAA. Lastly, Hal was compared with the effect of treatment with picloram (Pic) alone.

The effects of different concentrations of Hal and aminopyralid (AP) were tested on the hormone (IAA and ABA) levels in *B. napus* seedlings at two different temperatures or growth stages. Determination of the optimum Hal concentration revealed that already, the lower concentration altered the hormonal profile with respect to IAA (Figure 3A). Surprisingly, the free IAA levels decreased after 96 h of Hal treatments, while for ABA, there was no dramatic effect at this time point. This was observed for both concentrations of Hal. Therefore, in all experiments, the lower Hal concentration has been used in the treatments. Since the results for free IAA were the most interesting, these will be described in more detail in the following paragraphs.

Auxinic herbicides can cause typical hormone-like symptoms in oilseed rape dependent on temperatures in the field. Therefore, it was tested whether Hal was acting differently at 15 °C or 9 °C during the growth period. There was an influence of incubation temperature: at 15 °C, Hal-treated plants show levels of IAA significantly lower vs. untreated, while at 9 °C, the effect was not as pronounced. There was no reduction of IAA at lower temperatures. At 15 °C, AP seemed to increase the Hal effect. In conclusion, 96 h after Hal application, the IAA levels are significantly reduced at 15 °C (Figure 3B).

The concentration of Hal per plant was adjusted for the different growth stages BBCH14 compared to BBCH11. There was an influence of the development stage of *B. napus*: more developed Hal-treated plants show a lower level of IAA vs. untreated compared to smaller plants, while smaller plants tend to have increased the ABA level. This effect on IAA was consistently found at this time point and at the temperature of 15 °C. The analysis in separate tissues of the *B. napus* later during development showed that older leaves of these plants did not show the effect, while younger leaves of the same plants showed the reduction of IAA, which was also very prominent in the newly grown tissue. The addition of AP was neutral or increased tendencies slightly (Figure 4). The reduction of IAA by Hal was detectable for both growth stages. In the first experiment, in the total leaves of older plants, free IAA was more reduced than in younger plants (Figure 4A). The experiment was repeated, and for older plants, the different leaves were harvested separately (Figure 4B,C). Interestingly, the older leaf pair did not show the reduction in IAA, while the younger leaf pair and the newly formed plant material showed it. There seems to be a fine-tuned regulation in different tissues and also under different growth conditions for the regulation of IAA after auxinic herbicide treatment.

As already seen for the first experiment, stage BBCH11 showed a reduction of free IAA and stage BBCH14 showed only a reduction of IAA in the younger leaves and the newly grown material. It should be noted that the leaf pair 1 + 2 are older leaves, whereas the leaf pair 3 + 4 equals young tissue similar to leaf 1 from BBCH11. The newly grown material showed the highest effect, but in two control samples, hormone analysis was not possible. 

### 3.2. Time Course of Hormone Levels

In the first set of experiments, three different time points (24, 48, and 96 h) were analyzed for free IAA and ABA. All the following experiments were performed on leaves of the growth stage BBCH11 and a growth temperature of 15 °C. Sample values were always biological triplicates with two technical replicates for one data point. Additional individual data points for Hal treatment, shown as squares in the time course, were from additional experiments performed at different years (Figure 5A,B). Since AP did not increase the effects on IAA dramatically at the tested time points (data not shown), in that second experimental set, the co-treatment with AP was not further performed (Figure 5). In addition, Pic, the auxinic herbicide that is part of the commercial mixture with Hal, was used as an individual compound. 

Since auxinic herbicides do not only induce ABA, but also the synthesis of ethylene [5], the possibility of an increase in this hormone was also evaluated. ACC is the direct precursor of ethylene and often measured instead. We considered only changes in hormone levels reproducible when these were found in all independent experiments. ABA as a stress hormone did not change much over time, and the variation between experiments done at different seasons is high (Figure 5). IAA showed a different pattern over time with the two auxinic herbicides. It increased strongly after 4 h in the treatments with both auxinic herbicides, which was seen up to time point 72 h. At 96 h, the IAA decreased in Hal-treated samples but not in Pic-treated ones. This was less strongly observed at 120 h, while at the last time point (168 h), no differences were found any more between the two compounds. ACC was almost not present at 2 and 4 h after treatment with both compounds, but then a strong increase was observed, which was more pronounced after Hal treatment. The apparent decrease at the time point of 96 h was due to low values of treated samples but high controls (Figure 5C). When the data were calculated as percent of control, the decrease was not so strong any more (Figure 5C, second panel). Overall, the effects seen were transient for both herbicides, since after 168 h, there were only differences visible for ACC but not for IAA.

The conjugated IAA fraction compared to free was elucidated at two time points in a second experiment. The fraction of total IAA was determined after alkaline hydrolysis (see Section 2.2.3). It contains both, free and conjugated, IAA. The value for free IAA is subtracted from the value for the total, which then yields conjugated values. The conjugates measured by this method include both ester and amide conjugates [27]. The hydrolysis of IAA conjugates was also included successfully to the protocol (Figure S2), since IAA-Asp could be almost completely hydrolyzed, while the IAA added in the trial was not strongly degraded by the method. 

Both auxinic herbicides induced the amount of conjugated IAA compared to controls at 48 h and even more at 96 h (see Section 3.5). While the effect of Pic was somewhat stronger at the first time point, Hal induced the conjugated IAA levels much stronger than Pic at the second time point (96 h). This correlates with the decrease of free IAA found (Figure 5). 

### 3.3. Transcriptome Analysis after Treatment with Auxinic Herbicides

To elucidate the molecular background transcriptome, analyses were performed with control, Hal, and Pic-treated samples for three time points. Samples analyzed were control, Hal, and Pic after 2, 48, and 96 h based on the previous data of hormone concentrations. The hormone levels were similar in all sample types at 2 h, which was a reasoning to use this time point also in the transcriptome analysis as a “zero” point. After 48 h, the free IAA was upregulated, while at 96 h, a downregulation was found after Hal but not Pic treatments. Therefore, these two time points were chosen also for the transcriptome. 

The samples were processed by Novogene, which also performed the statistical and data analysis. The quality was very good for all samples and the number of reads obtained was similar (Tables S1 and S2). In Figure S3, the workflow for mRNA sequencing data of standard bioinformatic analysis with a well-annotated reference genome as with *B. napus* is shown. The number of differentially expressed genes increased over time for both herbicides, and the number of upregulated and downregulated genes was higher in Hal-treated leaves compared to Pic-treated ones (Figure S4).

For each treatment, three replicates were done. In the transcriptome analysis, the number of transcripts for the herbicide treatments was compared to the number of transcripts for the respective gene in the control sample, and a ratio was calculated using the mean values of the three replicates. This was calculated for each time point comparison. Overall, there were not so many genes differentially regulated at the early time point for both treatments compared to controls, while the number of differentially regulated transcripts increased over time. Hal treatment caused a stronger regulation than Pic. The data are shown together with the differential regulation of genes encoding conjugating enzymes (see Section 3.5).

**Figure 6:**
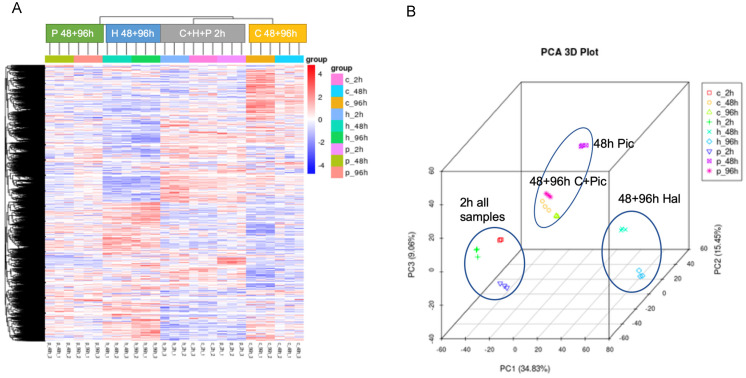
Clustering of samples. (**A**) Cluster of individual samples revealed that the replicates cluster together and also the three samples at the earlier time point (2 h) are more similar to each other that at later time points. The intensity of color shows the number of transcripts for that specific gene. (**B**). The PCA plot shows in principle the same trend for the clusters, but here, the Hal treatments after 48 and 96 h seem to be more separated. The figures were provided by Novogene and slightly altered. Abbreviations are ‘h’/‘H’ for Hal and ‘p’/‘P’ for Pic.

Among the statistical analyses performed, also a clustering of all sample sets is done (Figure 6). These show that the triplicates cluster tightly together, indicating a highly comparable replicate set (Figure 6A). All the treated samples clustered together at the 2 h time point (gray cluster). For later time points, the treatments (blue, green clusters) and controls (yellow cluster) were separated from each other. In the PCA plot, the later time points for Hal treatment are more separated from the rest (Figure 6B).

### 3.4. Evaluation of General Functional Groups

GO is the abbreviation of Gene Ontology (http://www.geneontology.org/), which is a major bioinformatics classification system to unify the presentation of gene properties across all species. It includes three main branches: cellular component, molecular function, and biological process. Through the enrichment analysis of the differential expressed genes, one can find out which biological functions or pathways are significantly associated with differential expressed genes. The differentially expressed genes were grouped into functional categories. A gene group that shows up has a significant number of genes differentially expressed in the specific group. 

**Figure 7:**
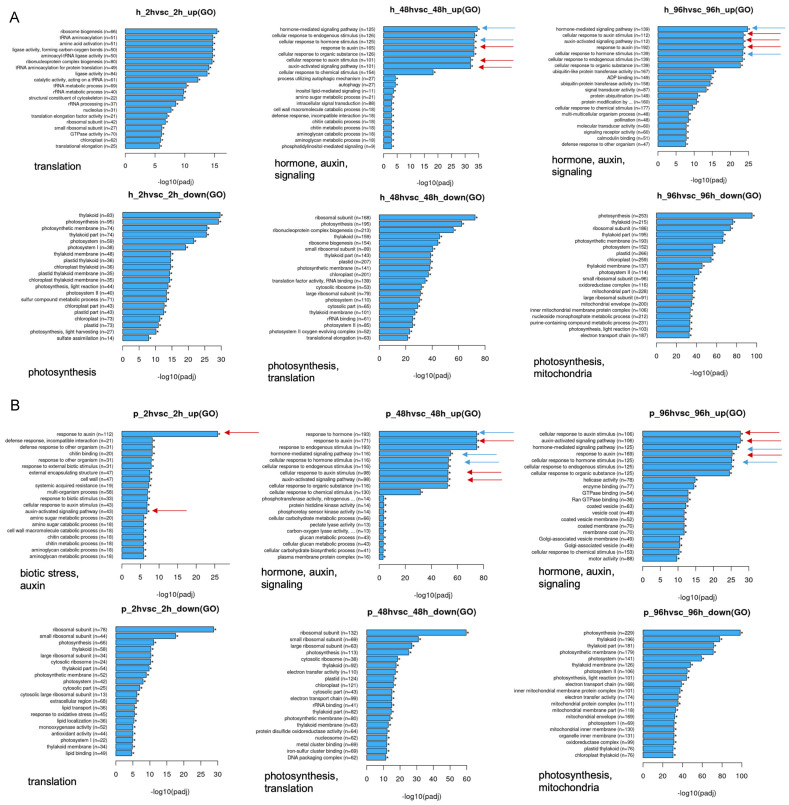
The significant enrichment of GO terms after Hal (**A**) and Pic (**B**) treatments at three different time points (2, 48, 96 h) compared to controls. The upper three panels show the upregulated genes, the three lower panels show the downregulated ones. Hormone-associated genes are indicated by blue arrows, auxin-related genes are indicated by red arrows. The major GO terms are indicated below each separate panel as a summary. The panels were supplied by Novogene. Abbreviations are ‘h’ for Hal and ‘p’ for Pic. GO terms with corrected *p*-value > 0.05 were considered significantly enriched by differential expressed genes and are marked by asterisks.

Analysis of GO (Gene Ontology) terms (Table S3) that indicate functional classes revealed different patterns for samples after Hal and Pic treatment. The top 20 significantly enriched terms in the GO enrichment analysis are displayed in Figure 7. There are interesting differences for the treatments. Hal treatment led to the upregulation of translational processes after 2 h, whereas auxin and hormone responses were upregulated later at 48 and 96 h. Photosynthesis and mitochondrial processes were downregulated through all time points. Furthermore, after 48 h, translation was downregulated (Figure 7A). Pic treatment induced the hormone/auxin response already after 2 h, and this continued for the other time points. At 2 and 48 h after treatment, the translational processes were downregulated, and photosynthesis was downregulated after 48 and 96 h (Figure 7B). In addition, a strong upregulation of biotic stress-associated processes was found after Pic treatment for the early time point, which was unexpected. At 2 and 48 h after treatment, the translational processes were downregulated, and photosynthesis was downregulated after 48 and 96 h in the Pic-treated plants. At the last time point of harvest, mitochondrial processes were downregulated, similarly as seen for Hal treatment.

**Figure 8:**
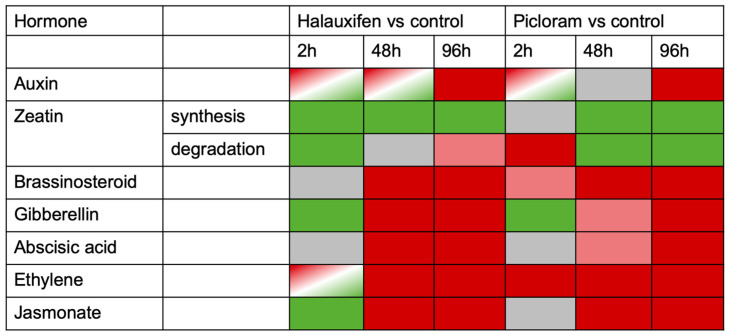
Differentially regulated KEGG pathways for Hal and Pic treatments after 2, 48, and 96 h compared to controls with respect to genes encoding hormonal biosynthetic pathways. Summary of regulation drawn as a heat map. Green = majority of genes downregulated; red/light red = majority of genes upregulated; red/green = some genes upregulated and some downregulated in the same pathway; gray = no regulation of any gene from one pathway. The complete pathways are shown as KEGG representations in Figure S6.

**Figure 9:**
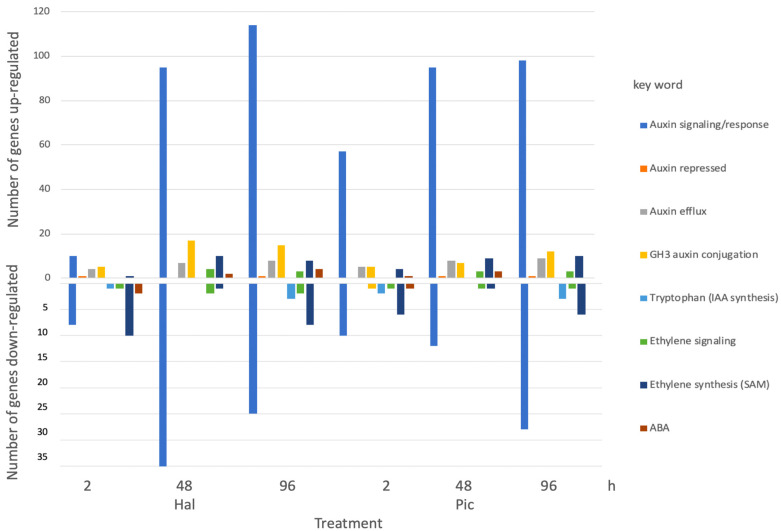
The number differentially expressed genes after 2, 48, and 96 h treatment with Hal and Pic compared to controls according to keyword search. As keywords auxin, IAA, tryptophan (as IAA synthesis precursor), ethylene, SAM, and ACC (as ethylene synthesis precursor), as well as ABA were used. Neither ‘IAA’ nor ‘ACC’ as a search term were found.

Additional evaluations have been done using the KEGG database (Kyoto Encyclopedia of Genes and Genomes, http://www.kegg.jp/) to identify differentially regulated pathways (Figure S5) [30]. This is a collection of manually curated databases containing resources on genomic, biological pathway, and disease information. Pathway enrichment analysis identifies significantly enriched metabolic pathways or signal transduction pathways associated with differentially expressed genes, comparing the whole genome background. This function is different from the GO annotation, since it results in the significantly regulated pathways that have been annotated. These pathways include 117 individual ones for all comparisons separated into up- and downregulated genes and are for primary metabolism, secondary metabolism, hormones, signaling, transport, cellular functions, etc. The KEGG database also results in pathway pictures, where the differentially expressed genes are labeled with different colors according to their expression level (Figure 8 and Figure S6). A heat map has been constructed from such individual panels (Figure S6) to give an overview on the hormonal effects (Figure 9). Then, individual pathways have been extracted for each of the plant hormones (brassinosteroid, cytokinin-specifically zeatin, auxin, gibberellin, ABA, ethylene and jasmonate), and the respective pathways are shown in the supplement (Figure S6). 

### 3.5. Evaluation of Hormone-Related Genes in More Detail

A search for different hormone-related keywords resulted in the number of up- and downregulated genes in each treatment (Figure 9). Specifically, the terms for auxin signaling/response, auxin transport, tryptophan (for auxin synthesis), ethylene signaling and synthesis (search term S-adenosyl-methionine, SAM and ACC, but the latter was not found as search term), and ABA synthesis and signaling were applied to excel files with all up- and downregulated genes for each comparison. Within the lists, the number of genes was counted and added up. The genes encoding auxin-conjugating proteins were not found by this method; they were retrieved using the gene numbers supplied by [29] on *B. napus GH3* gene families. The majority of auxin-related genes are grouped into signaling and also auxin efflux. Among the former groups, there could be genes encoding auxin receptors, auxin transcriptional repressors and activators as well as auxin-responsive genes. These categories would have to be analyzed further by checking individual gene numbers. In this category, both herbicide treatments resulted in similar patterns, namely more genes were up- than downregulated. Furthermore, a group of auxin efflux carriers was upregulated in all treatments, but there were no differences in the numbers of these between treatments. The latter might point to a role in decreasing auxin levels from the cells, and the plants treated with an auxinic herbicide might induce the efflux machinery to get rid of too high auxin levels. The synthesis of tryptophan as an IAA precursor was not differentially regulated, but as shown by KEGG pathways, genes encoding proteins in auxin biosynthesis are indeed differentially regulated, and they are mainly upregulated (Figure 8 and Figure S6). In the KEGG data, it is not possible to get an indication of how many genes are regulated; only significant differences in complete pathways are shown, and within these, individual gene regulations can be displayed [30]. 

Searching for ethylene or the precursor for biosynthesis (SAM) showed some ethylene-responsive genes and also a few genes encoding proteins involved in ethylene biosynthesis, but these were not only upregulated; there were similar numbers downregulated (Figure 9). The KEGG data showed that more ethylene synthesis genes are up- rather than downregulated (Figure 9 and Figure S6). ABA-related genes were not much expressed, as it was already suggested by the small response of ABA levels in the different treatments; albeit, there was some regulation again occurring in the KEGG pathways. 

**Figure 10:**
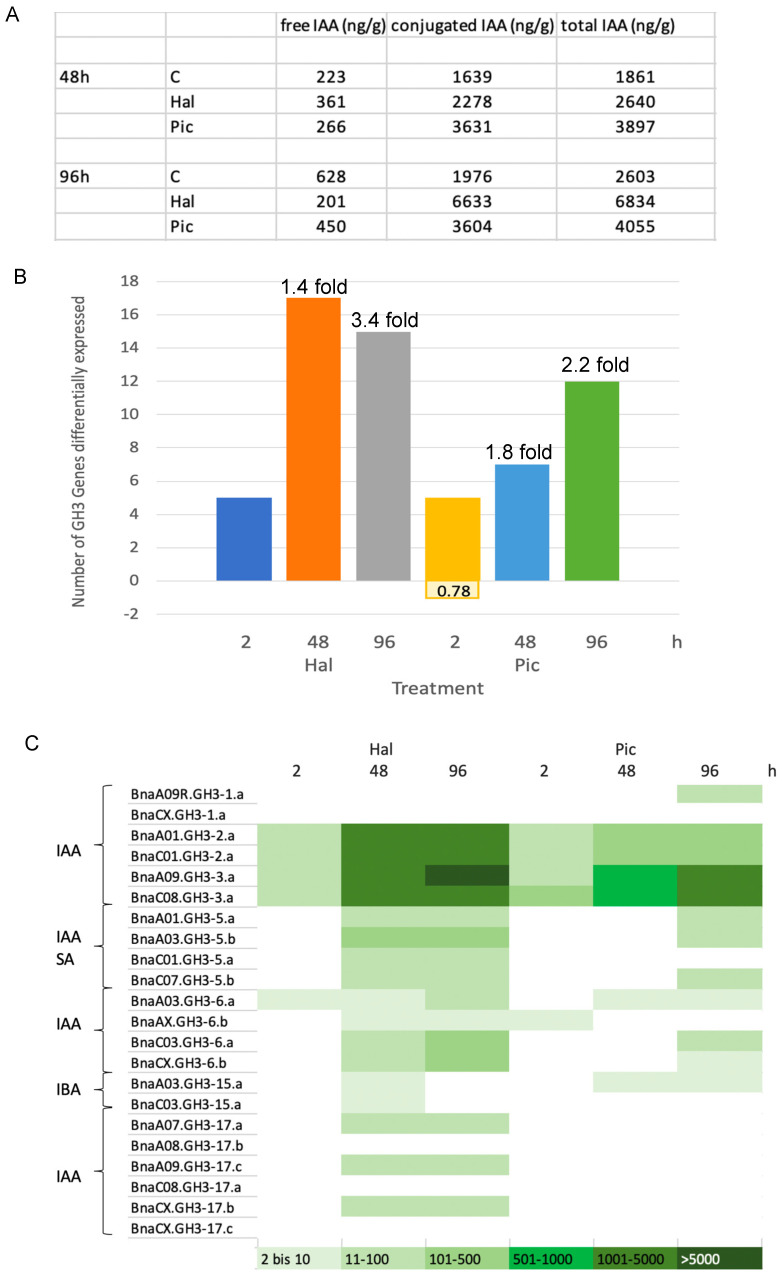
(**A**) The levels of free and total/conjugated IAA at 48 and 96 h after treatment with the two auxinic herbicides Hal and Pic. (**B**) The number of auxin-conjugating *GH3* genes differentially expressed. The values for the ratios as well as mean + SD can be found in Table S4. The numbers on top of the histograms show the x-fold increase of conjugated IAA in the treated sample vs. control (see for numbers panel A). (**C**) The relative expression levels of auxin-conjugating *GH3* genes in treatments compared to controls. The calculations were done manually by using the mean values of treated samples vs. controls, and then ratios were calculated for treatment H (Hal) and P (Pic) vs. control and placed into classes. On the left, the substrates are given that are verified for *A. thaliana*. IAA = indole-3-acetic acid; IBA = indole-3-butyric acid; SA = salicylic acid.

Since the auxin conjugation seems to play a role in the hormone phenotype of Hal and Pic-treated plants, the *GH3* genes encoding auxin amino acid synthetases were manually curated (Figure 9 and Figure 10, Table S4). Among these, there was an increase in the number of *GH3* transcripts upregulated over time, but there were also differences between the two auxinic herbicides, and Hal gave a stronger upregulation of this gene family than Pic. The upregulation of the *GH3* genes correlates with the increase in conjugated IAA at the same time points and treatments (Figure 10A,B). The numbers on top of the histograms (Figure 10B) show the x-fold increase of conjugated IAA in the treated sample vs. controls (Figure 10A). Only one *GH3* gene was found to be downregulated in the 2 h treatment with Pic, but the ratio between controls and treatment was low, so that one cannot assume a differential regulation.

The annotation of the *GH3* genes is based on what is known in the model plant *A. thaliana* and was taken from [29] for *B. napus*. The ratio for each *GH3* gene was calculated by using the mean value for Hal or Pic expression and the controls at the respective time point. Then, the mean value of the treatment was divided by the mean value for the control, and categories were made. Below a 2-fold induction, it was not considered to be a significant differential regulation. Therefore, the first category starts with 2- to 10-fold. 

The number of genes reflect (a) the duplication events as compared to *A. thaliana* and (b) the two different genomes (A and C genome) present in the hybrid *B. napus* [29]. The substrates given on the left have only been verified for this plant species and not for *B. napus* or other brassicas. The *GH3.2* and *GH3.3* types were highly upregulated in both treatments over the time (Figure 10C). However, for Hal treatment, more *GH3* genes were regulated, and also, a stronger regulation level was found. This correlates with the findings that free IAA is downregulated and conjugated IAA is upregulated at the same time points. The gene encoding the GH3.5 protein, which also conjugates salicylic acid (SA) to amino acids, is moderately upregulated. The gene encoding GH3.6 is more upregulated than *GH3.5*, *GH3.15*, and *GH3.17* which are only regulated after Hal, but not Pic treatments (Figure 10C), and there are indications that GH3.6 might be involved in the abiotic stress response of *A. thaliana*. Whether IBA conjugates play a role in *B. napus* has not been shown yet. Other *GH3* genes with substrates not connected to IAA are not shown here.

## 4. Discussion

### 4.1. Hormonal Phenotype of B. napus Treated with Auxinic Herbicides under Different Growth Conditions

Synthetic auxins are often used as herbicides due to their capability to inhibit growth at higher concentrations. The events occurring after treatments with auxinic herbicides have been investigated and were attributed to changes in ethylene and ABA by increased auxin-induced gene expression (Figure 1). While the role of ethylene and ABA have been already found in the last decades to be connected to the growth phenotype [5], more recent work suggested a participation of ABA only [16]. However, the levels of IAA have not been documented as detailed, as done for ABA. Here, it was shown that the treatment with three different auxinic herbicides (Figure 2) resulted in sometimes similar responses, but details were different between the compounds such as levels of hormones and the number of differentially expressed genes in the categories observed. The hormone-related Hal phenotype can be attributed to the increased ethylene synthesis as well as altered auxin levels by conjugation, but more research is necessary to confirm this; for example, the determination of enzymatic activities of the GH3 proteins from *B. napus* and a more detailed analysis of the gene dataset. 

Often, herbicides are applied as preformulated mixtures, which reduces the probability of resistances [31,32]. In the case of the two auxinic herbicides investigated here, halauxifen-methyl and picloram are present as a mixture in commercial preparations [12]. This commercial compound is a broad-spectrum broadleaf herbicide for autumn use on oilseed rape [12]. In order to understand better the influence of two auxinc herbicides on the crop response, it was therefore of interest to investigate the biochemical and molecular phenotype of this phenomenon in *B. napus*. In addition, the effect of different concentrations of Hal and a second auxinic herbicide (AP) were tested on the hormone (IAA and ABA) levels in *B. napus* seedlings at two different temperatures or growth stages. Based on the investigations on hormones that respond to the auxinic herbicides [5], the levels of IAA, ABA, and ACC were determined over time. It was shown that ABA did not show a consistent trend in different experimental setups, but free IAA was reduced specifically at certain conditions. The effect could be attributed to growth at 15 °C and the younger leaves of two different developmental stages, but it was not further increased in combination with AP. However, a time course indicated that the elevation of IAA is specific to earlier time points after treatment (Figure 5), starting 2 h after treatment, while specifically 72–120 h after treatment, a decrease in free IAA was found. The observation could also be made for a second auxinic herbicide, Pic, but it was not as prominent. 

A possible contradiction seen between the ABA levels that did not change significantly over the time points (Figure 5), and the observed upregulation of ABA biosynthesis-related genes (Figure 8 and Figure S6) could be probably explained by (i) that we determined only free ABA and that ABA can also be conjugated (especially to glucose [33]) or degraded to (dihydro)-phaseic acid [34] and (ii) that the plant material used for RNAseq showed indeed an upregulation (albeit with high statistical variation), but this was not reproducible within experiments carried out at different seasons. (iii) ABA as a stress hormone may be additionally more affected by micro-stress factors in individual experiments.

ACC, as a precursor for ethylene, increased in almost all time points for both auxinic compounds, but the increase was more pronounced for Hal. A very recent study compared ABA and ethylene levels in a weed species that was also treated with Hal [16]. While the crop did not show reproducible alterations in ABA levels for the two auxinic herbicides applied (Figure 5), the weed showed a strong increase in ABA in treated leaves not 1 but 6 h after treatment, which continued over the next few days. Furthermore, the ABA increase was also observed for two other auxinic herbicides, 2,4-D and dicamba. This was accompanied by increased levels of genes encoding biosynthesis enzymes. No reproducible evolution of ethylene was reported after Hal treatment [16], while in our work, ACC as an ethylene precursor increased dramatically, more after Hal than Pic treatments over a longer time period (Figure 5).

High IAA might be toxic and growth inhibitory for the plant, which is also true for auxinic herbicides, the latter being more stable inside the plant. Since the concentration of IAA decreased in treated leaf samples after longer incubation times, it was hypothesized that IAA could be conjugated to form inactive compounds. One major form of conjugates are the amino acid conjugates [3] that are enzymatically synthesized by the family of so-called GH3 proteins [35]. This reaction leads to the formation of amino acid conjugates, which can either be enzymatically hydrolyzed back to free IAA or, in case of IAA-aspartate and IAA-glutamate, be enzymatically degraded [3]. In *A. thaliana*, it was shown by microarray and qPCR analysis that one *GH3* gene (*GH3.3*) was strongly upregulated after treatment with the auxinic herbicide dicamba [4]. However, another hypothesis was that the auxinic herbicides are not substrates for the GH3 proteins and thereby stay in the tissue for a longer time [7]. In resistant plants, the GH3 proteins could then convert the auxinic compound to amino acid conjugates and would thereby minimize the growth-retarding effects. However, there is one report on the conjugation of dicamba to glutamate by some plant enzymes [8] as well as for 2,4-D and 2,4 dichlorobutyric acid [36], but these are only in vitro data, and the situation in planta might be different. 

After Hal treatment, we found a decrease in free IAA, which would fit into the scenario that Hal could induce *GH3* gene expression but would itself not be conjugated. For the first assumption, we have shown that the pool of IAA conjugates is upregulated together with a strong upregulation of several auxin-conjugating *GH3* genes in *B. napus*. Whether this takes place without conjugating Hal to amino acids has yet to be tested. In vitro enzyme assays would of course be possible, but with the respective enzymes from *B. napus*, the number that needs to be tested is large. *GH3* genes belong to the group of auxin-responsive genes [37]. Some authors have shown that not only IAA but also some auxinic herbicides increased IAA conjugation [4,6,9]. In addition, some chlorinated IAA derivatives can be conjugated by at least one GH3 protein family member of *A. thaliana* [38], indicating that the substrate specificity toward auxins might be broader that originally anticipated [37]. 

### 4.2. Molecular Phenotype of B. napus Treated with Auxinic Herbicides

A transcriptome analysis was carried out for three time points after treatment to determine changes in the complete transcriptome, but the data were screened with an emphasis on auxin (conjugation), ethylene, and ABA. The results showed that the ethylene precursor ACC was strongly upregulated during the course of the experiment in both auxinic herbicide treatments (Figure 5), while there was again no trend for the stress marker ABA. The treatment with auxin and auxinic herbicides could result in an increase in ethylene, since one of the genes encoding one enzyme in the pathway (ACC synthase) is also auxin-regulated (Figure 2) [5]. Ethylene, in turn, could alter the growth phenotype to this epinastic bending [39]. More general effects that we observed for both auxinic herbicides were the differential regulation of pathways with connection to protein biosynthesis and the downregulation of photosynthesis and mitochondrial processes (Figure 7). A downregulation of genes associated with photosynthesis was also found for the weed *E. canadensis* for all three herbicide treatments [16]. 

Albeit the role of the GH3 proteins in this process is not clear, many *GH3* genes that encode proteins involved in auxin conjugation were upregulated (Figure 8 and Figure 9). After 2 h, there was a low number of *GH3* genes for both auxinic herbicides, whereas the number of *GH3* genes upregulated was higher after Hal treatment 48 and 96 h after application than for Pic. Since the annotation of the *GH3* genes is based on what is known from *A. thaliana*, it cannot be ruled out that additional phenotypes may play a role in the phenotype observed after treatment. Strong growth retardation phenotypes for dominant mutations in two *GH3* genes (*GH3.5* and *GH3.6*) have been shown [40,41], which partially resemble the ones after auxinic herbicide treatments. *GH3.5* homologs are also strongly upregulated in the transcriptome after herbicide treatments (Figure 10). In addition, *GH3.6* genes are upregulated, which have been indicated to play a role in abiotic stress responses in plants [40,41]. This could be an indication that also in *B. napus*, the herbicide treatments result in a stress response, but the substrates for the *GH3* genes mentioned have yet to be confirmed. So far, in the literature, only transcriptome data for 2,4-D and dicamba are available in the model organism *A. thaliana* [4,6]. In addition, a *Pisum sativum* auxin conjugate synthetase was upregulated by IAA, 2,4-D and the two auxinic herbicides dicamba and Pic on the gene expression and enzymatic level [9]. As for auxin perception and signaling, the highest number of genes differentially regulated was found (Figure 9). It was shown that two out of six auxin receptor proteins are able to perceive picloram [11]. The further analysis of this large functional group will be of interest to find which specific pathways for auxin are regulated after Hal and Pic treatments. For the GH3.5 protein of *A. thaliana*, the conversion of SA to amino acid conjugates was reported [42], but whether this substrate specificity plays a role for the biochemical and molecular auxinic herbicide phenotype has not been established. 

Other growth-related hormones, such as brassinosteroids (BR), gibberellins (GA), IAA, and cytokinins were also differentially regulated in their biosynthetic pathways (Figure 8 and Figure S6). The upregulation of BR pathways could also result in altered growth phenotypes, since they regulate elongation growth and cell expansion as auxins [43,44], but to our knowledge, there was no connection yet made so far with auxinic herbicides. However, there is an observation that BR can inhibit the growth of etiolated pea seedlings by increasing ethylene production, and this effect is stronger than the one after IAA treatment [45]. Such experiments suggest that also the induction of BR biosynthesis (Figure S6A) by auxinic herbicide treatment could contribute to the increased ethylene production, as was estimated by the high ACC levels that were found (Figure 5). The high ACC levels in treated plants were backed up by the observed increase in the upregulation of the ethylene pathway for all treatments (Figure S6F). In addition, BR and IAA can also induce GA biosynthesis [46,47,48,49]. Since GA biosynthesis was also more upregulated for the time points 48 and 96 h, but downregulated 2 h after treatments (Figure S6D), it could also be involved in the alteration of growth phenotypes. Interestingly, Hal treatment more strongly upregulated GA biosynthesis genes than Pic. Zeatin (a cytokinin) was downregulated in its synthesis, but one gene encoding a degradation enzyme was upregulated. This enzyme catalyzes the reaction from dihydrozeatin to the respective O-glucoside by UGT85A1 [50]. The other noted gene is now known to glycosylate in the BR synthesis pathway [51] and the other one is now known to glycosylate in steviol [51]. Since also the ratio between auxin and cytokinin plays a role in altered phenotypes [52], this observation might be important as well. 

Hormones involved in the stress response of plants in addition to ethylene, namely ABA and jasmonate (Figure S6E,G), were also differentially regulated in their biosynthetic pathways. ABA synthesis was upregulated in both treatments 48 and 96 h, but this was not reflected in the hormonal contents (Figure 5). The jasmonate metabolism related genes were not regulated in a uniform manner. No indication for a regulation of the biosynthesis of SA has been found, but the current knowledge of the SA pathway might not yet have been annotated.

## 5. Conclusions

Auxinic herbicides inhibit the growth of their target plants in the fields, but they can occasionally also modulate the growth of a respective crop plant. *B. napus*, being such an important crop, was used to address the following questions. What is the impact of the temperature and the growth stage at which the auxinic herbicides cause changes in a selected group of plant hormones, and what is the impact of individual herbicides or a combination that is used in the field? In these experiments, not only one auxinic herbicide was used, but we compared two that are relevant for practical approaches. Finally, an important factor is the time after which plants would react to the treatments. Our approach is novel, since it does not use ambient temperature conditions but mimics the growth conditions in the field at the time of application in late summer/autumn. The hormone response resulted specifically for the 15 °C treatment in a change in IAA and ACC levels, but ABA was not reproducibly altered. Further transcriptome analyses corroborated the findings from hormone measurements. Indeed, auxin-related genes, mainly signaling and transport but also the conjugation to amino acids was differentially regulated. Genes encoding such GH3 proteins were strongly upregulated. In addition, other growth hormone pathways were also upregulated as well as the pathways for ethylene and ABA. In conclusion, this hormone and transcriptome dataset sheds new light on the processes involved in the auxinic herbicide response in *B. napus*, since it also compares two different auxinic herbicides. Further evaluation of the groups of differentially expressed genes related to other processes, such as stress or metabolic events, which have not been addressed here, can be used in the future to further shed light on the processes described. In this context, a verification of selected differentially expressed genes is also needed.

## Figures and Tables

**Table 1:** Overview on the treatments with different formulations and combinations of auxinic herbicides. Hal = halauxifen-methyl; AP = aminopyralid; Pic = picloram.

Solution	Control	Hal	Hal + AP	Pic
DMSO	1%	1%	1%	1%
Triton X-100	0.01%	0.01%	0.01%	0.01%
NaOH	0.01 M	0.01 M	0.01 M	0.01 M
Hal	-	0.1 g/L	0.1 g/L	-
AP	-	-	0.36 g/L	-
Pic	-	-	-	0.48 g/L

## Data Availability

Additional data are available in the supplement. The transcriptome data presented in this publication have been deposited in NCBI’s Gene Expression Omnibus [53] and are accessible through GEO Series accession number GSE179676 (https://www.ncbi.nlm.nih.gov/geo/query/acc.cgi?acc=GSE179676).

## Supplementary Materials

The following are available online at https://www.mdpi.com/article/10.3390/biom11081153/s1, Supplementary Methods for RNAseq, Figure S1: The two different growth stages BBCH11 and BBCH14 and the respective application points for auxinic herbicides, Figure S2: Method for auxin hydrolysis and confirmation of standard chromatograms, Figure S3: Workflow for gene expression analysis done by Novogene on the dataset, Figure S4: The number of differentially regulated genes after treatment with halauxifen-methyl and picloram over time (2, 48, 96 h) and Volcano plots, Figure S5: The interactions of multiple genes may be involved in certain biological functions, Figure S6: Differentially regulated KEGG pathways for halauxifen-methyl and picloram treatments after 2, 48, and 96 h compared to controls with respect to hormonal pathways, Table S1: Data quality summary as provided by Novogene, Table S2: Mapping of reads, Table S3: GO is the abbreviation of Gene Ontology (http://www.geneontology.org/), which is a major bioinformatics classification system to unify the presentation of gene properties across all species.
