# Supplementary material for: Two Auxinic Herbicides Affect Brassica napus Plant Hormone Levels and Induce Molecular Changes in Transcription
Source: Biomolecules. 2021 Aug 4;11(8):1153. doi: 10.3390/biom11081153 (PMC8391463; doi:10.3390/biom11081153)
Supplement: Supplementary file 1 [file biomolecules-11-01153-s001.zip › biomolecules-1312261-supplementary.pdf]

**Supplement to**

# Two auxinic herbicides affect *Brassica napus* hormone levels and molecular changes in transcription

Jutta Ludwig-Müller[1], Roman Rattunde[1], Sabine Rößler[1], Katja Liedel[1], Freia Benade[1], Agnes Rost[2], Jörg Becker[2]

[1]Technische Universität Dresden, Institut für Botanik, 01062 Dresden, Germany

[2]Corteva Agriscience Germany GmbH, Riedenburger Str. 7, 81677 München, Germany

[#]corresponding author: jutta.ludwig-mueller@tu-dresden.de

**Supplementary Methods for RNAseq**

The following text was directly taken without changes from the methods supplied by Novogene.

**RNA quantification and qualification**

RNA degradation and contamination was monitored on 1% agarose gels. RNA purity was checked using the NanoPhotometer® spectrophotometer (IMPLEN, CA, USA). RNA integrity and quantitation were assessed using the RNA Nano 6000 Assay Kit of the Bioanalyzer 2100 system (Agilent Technologies, CA, USA).

**Library preparation for transcriptome sequencing**

A total amount of 1 µg RNA per sample was used as input material for the RNA sample preparations. Sequencing libraries were generated using NEBNext® UltraTM RNA Library Prep Kit for Illumina® (NEB, USA) following manufacturer's recommendations and index codes were added to attribute sequences to each sample. Briefly, mRNA was purified from total RNA using poly-T oligo-attached magnetic beads. Fragmentation was carried out using divalent cations under elevated temperature in NEBNext First Strand Synthesis Reaction Buffer (5X). First strand cDNA was synthesized using random hexamer primer and M-MuLV Reverse Transcriptase (RNase H-). Second strand cDNA synthesis was subsequently performed using DNA Polymerase I and RNase H. Remaining overhangs were converted into blunt ends via exonuclease/polymerase activities. After adenylation of 3' ends of DNA fragments, NEBNext Adaptor with hairpin loop structure were ligated to prepare for hybridization. In order to select cDNA fragments of preferentially 150~200 bp in length, the library fragments were purified with AMPure XP system (Beckman Coulter, Beverly, USA). Then 3 µl USER Enzyme (NEB, USA) was used with size-selected, adaptor ligated cDNA at 37 °C for 15 min followed by 5 min at 95 °C before PCR. Then PCR was performed with Phusion High-Fidelity DNA polymerase, Universal PCR primers and Index (X) Primer. At last, PCR products were purified (AMPure XP system) and library quality was assessed on the Agilent Bioanalyzer 2100 system.

**Clustering and sequencing**

The clustering of the index-coded samples was performed on a cBot Cluster Generation System using PE Cluster Kit cBot-HS (Illumina) according to the manufacturer's instructions. After cluster generation, the library preparations were sequenced on an Illumina platform and paired-end reads were generated.

## Data Analysis

*Quality control*

Raw data (raw reads) of FASTQ format were firstly processed through fastp. In this step, clean data (clean reads) were obtained by removing reads containing adapter and poly-N sequences and reads with low quality from raw data. At the same time, Q20, Q30 and GC content of the clean data were calculated. All the downstream analyses were based on the clean data with high quality.

*Mapping to reference genome*

Reference genome and gene model annotation files were downloaded from genome website browser (NCBI/UCSC/Ensembl) directly. Paired-end clean reads were mapped to the reference genome using HISAT2 software. HISAT2 uses a large set of small GFM indexes that collectively cover the whole genome. These small indexes (called local indexes), combined with several alignment strategies, enable rapid and accurate alignment of sequencing reads.

*Novel gene prediction*

Because transcriptome annotations are still incomplete, most RNA-seq studies will reveal novel genes and transcripts. The Stringtie was used to assemble the set of transcript isoforms of each bam file obtained in the mapping step. gffcompare can compare Stringtie assemblies to reference annotation files and help sort out new genes from known ones.

*Quantification*

Feature counts was used to count the read numbers mapped of each gene, including known and novel genes. And then RPKM of each gene was calculated based on the length of the gene and reads count mapped to this gene. RPKM, Reads Per Kilobase of exon model per Million mapped reads, considers the effect of sequencing depth and gene length for the reads count at the same time, and is currently the most commonly used method for estimating gene expression levels.

*Differential expression analysis*

(For DESeq2 with biological replicates) Differential expression analysis between two conditions/groups (three biological replicates per condition) was performed using DESeq2 R package. DESeq2 provides statistical routines for determining differential expression in digital gene expression data using a model based on the negative binomial distribution. The resulting P values were adjusted using the Benjamini and Hochberg's approach for controlling the False Discovery Rate (FDR). Genes with an adjusted P value < 0.05 found by DESeq2 were assigned as differentially expressed. (For edgeR without biological replicates) Prior to differential gene expression analysis, for each sequenced library, the read counts were adjusted by Trimmed Mean of Mvalues (TMM) through one scaling normalized factor. Differential expression analysis of two conditions was performed using the edgeR R package. The P values were adjusted using the Benjamini and Hochberg methods. Corrected pvalue of 0.005 and |log2 (Fold Change)| of 1 were set as the threshold for significantly differential expression.

*Enrichment analysis*

A common way for searching shared functions among genes is to incorporate the biological knowledge provided by biological ontologies. Gene Ontology (GO) annotates genes to biological processes, molecular functions, and cellular components in a directed acyclic graph structure, and Kyoto Encyclopedia of Genes and Genomes (KEGG) annotates genes to pathway.

GO enrichment analysis: Gene Ontology (GO) enrichment analysis of differentially expressed genes was implemented by the clusterProfiler R package, in which gene length bias was corrected. GO terms with corrected Pvalue less than 0.05 were considered significantly enriched by differential expressed genes.

KEGG Pathway enrichment analysis: KEGG is a database resource for understanding high-level functions and utilities of the biological system, such as the cell, the organism and the ecosystem, from molecular level information, especially large-scale molecular datasets generated by genome sequencing and other high-through put experimental technologies (http://www.genome.jp/kegg/). We used clusterProfiler R package to test the statistical enrichment of differential expression genes in KEGG pathways.

*Alternative splicing analysis*

Alternative splicing analysis was performed by the software rMATS, a statistical method for robust and flexible detection of differential AS from replicate RNA-Seq data. It identifies

alternative splicing events corresponding to all major types of alternative splicing patterns and calculates the P value and FDR for differential splicing. These types include exon skipping (SE), alternative 5¢ splice sites (A5SS), alternative 3¢ splice sites (A3SS), mutually exclusive exons (MXE), and retained introns (RI).

*Mutation analysis*

Firstly, Picard tools and Samtools were used to sort, mark duplicated reads and reorder the bam alignment results of each sample. Then the tool HaplotypeCaller in GATK software was used to perform variant discovery. Raw VCF files were filtered with GATK standard filter method and other parameters (cluster: 3; WindowSize: 35; QD < 2.0 or FS > 30.0). Finally, ANNOVAR was used to functionally annotate genetic variants detected from diverse genomes with user-specified versions of genome builds.

## Supplementary figures

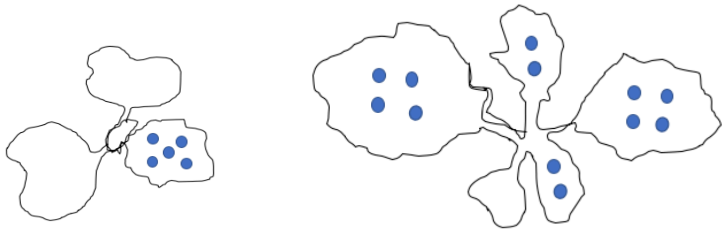

**Figure S1.** The two different growth stages BBCH11 (left) and BBCH14 (right) and the respective application points for auxinic herbicides (Halauxifen-methyl tech: 0,5 µg ai/plant; Picloram tech: 2,4 µg ai/plant; Controls were treated with the solvent only).

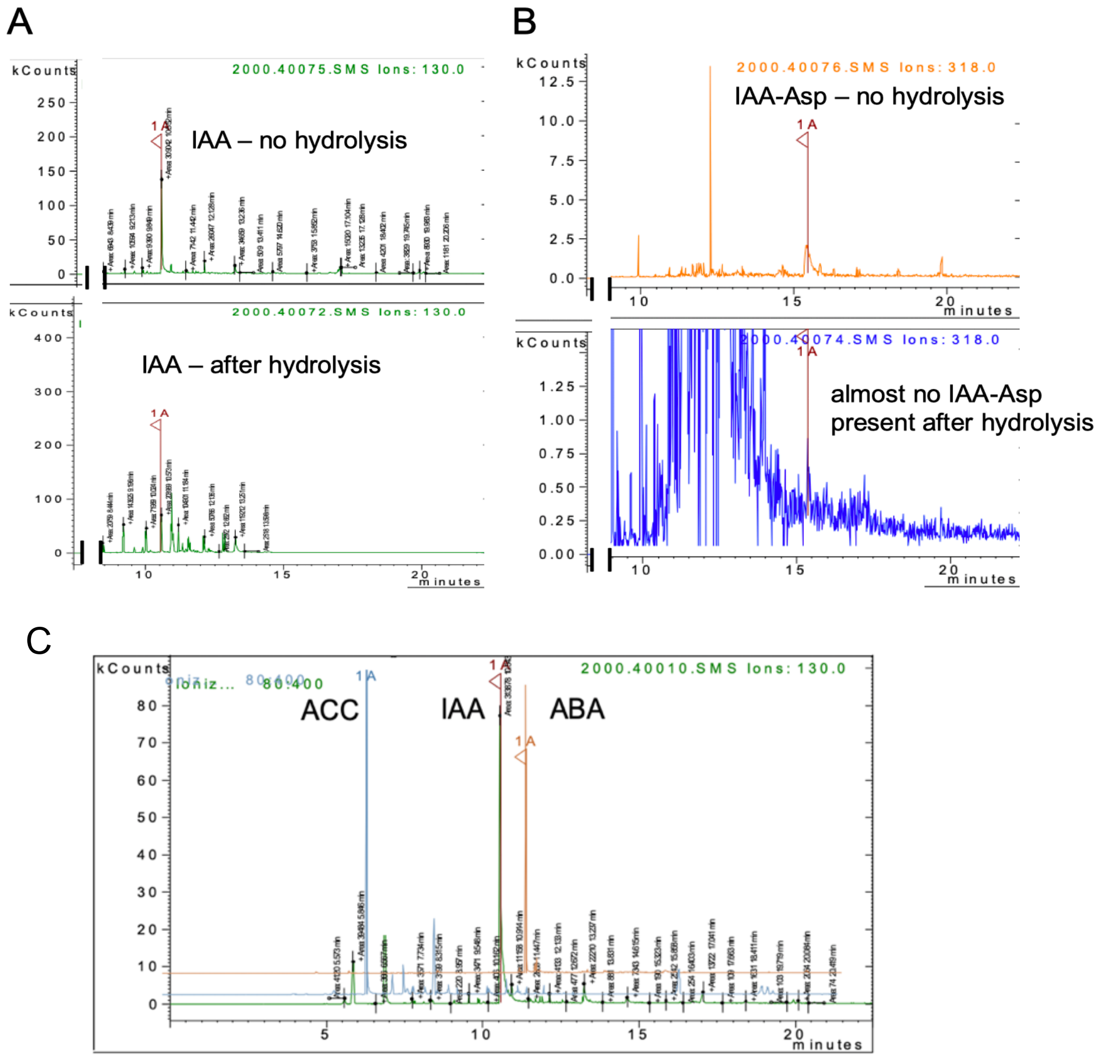


**Figure S2.** Method for auxin hydrolysis (A, B) after the hormone extraction based on [25,26] and confirmation of standard chromatograms (C). The alkaline hydrolysis (7N NaOH, 3 h at 90°C under $N_2$) of samples with IAA (A) and IAA-Asp (B) added.

A

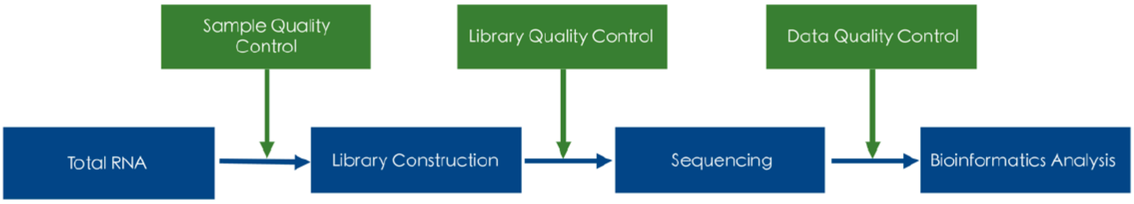


B

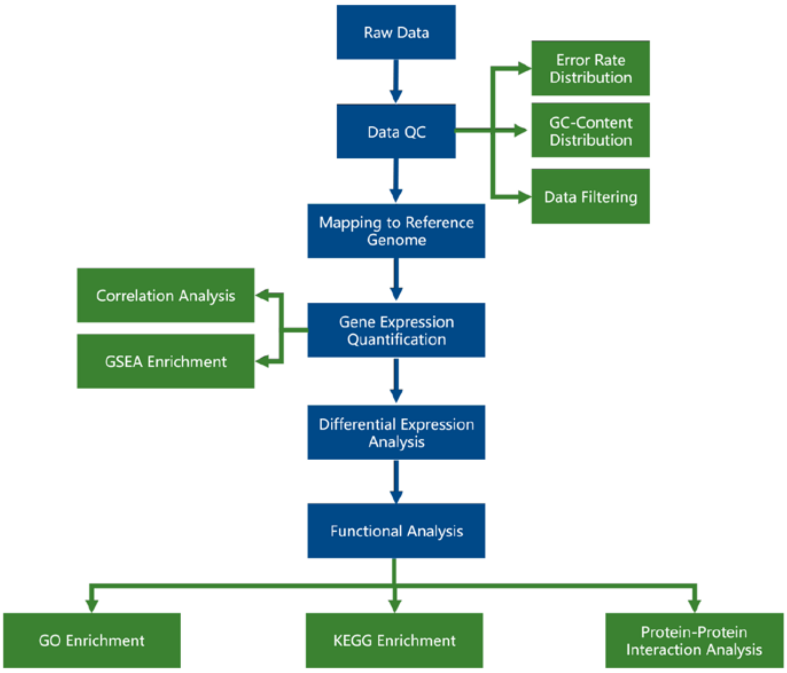


Novogene

**Figure S3** Workflow for gene expression analysis done by Novogene on the dataset. (A) protocol for sequencing. (B) Bioinformatics analysis.

A

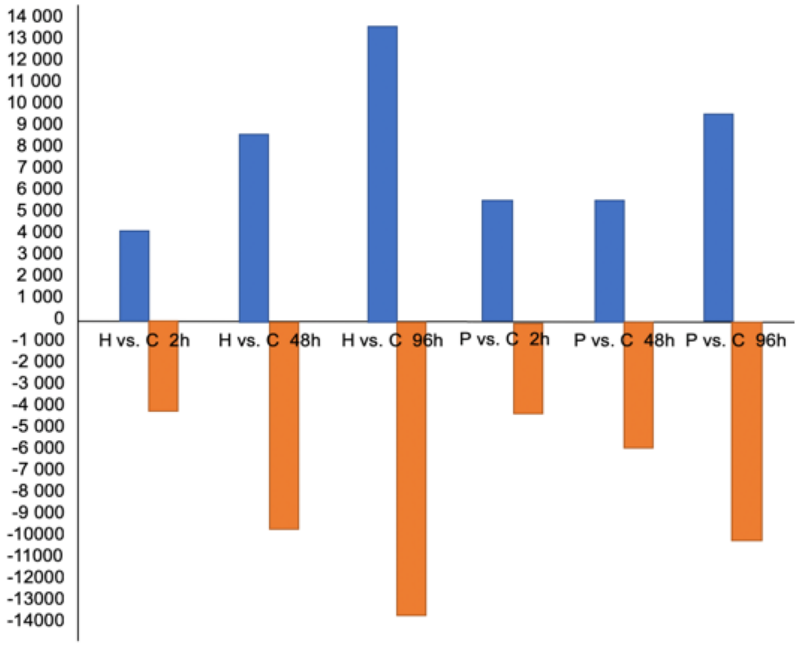


B

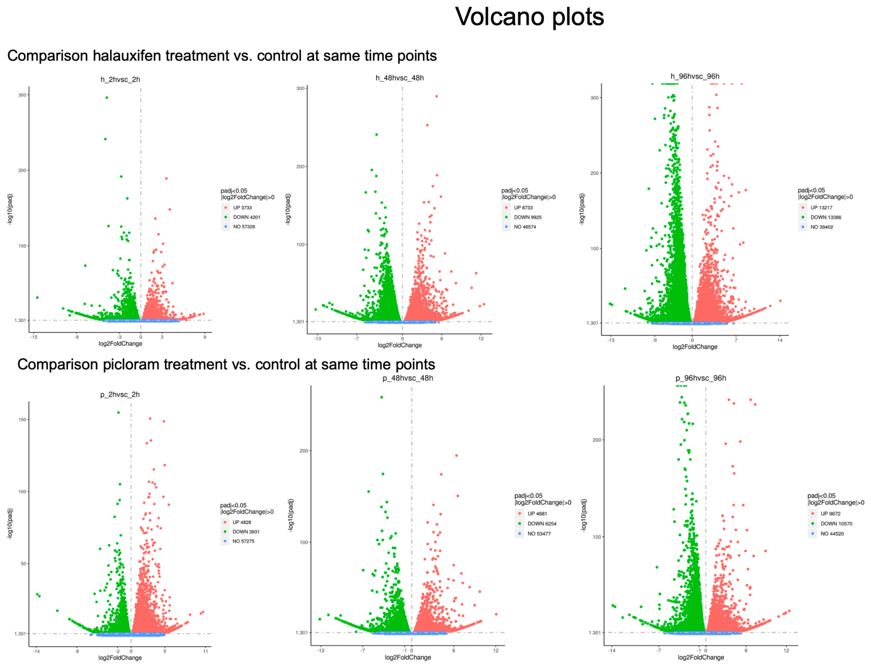


**Figure S4.** (A) The number of differentially regulated genes after treatment with halauxifen (h/H) and picloram (p/P) over time (2h, 48h, 96h). Both, up- and down-regulated genes increase over time. (B) Volcano plots where the x-axis shows how strong the genes are differentially regulated and the y-axis shows the significance of their regulation, the higher up a dot, the higher is the statistical significance.

$$P = 1 - \sum_{i=0}^{m-1} \frac{\binom{M}{i}\binom{N-M}{n-i}}{\binom{N}{n}}$$

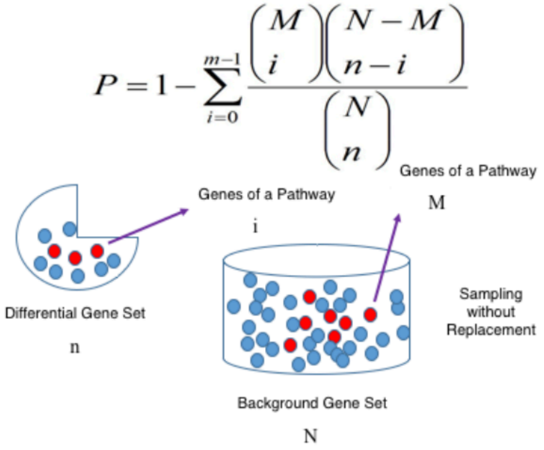


**Figure S5.** The interactions of multiple genes may be involved in certain biological functions. KEGG (Kyoto Encyclopedia of Genes and Genomes, http://www.kegg.jp/) is a collection of manually curated databases containing resources on genomic, biological-pathway and disease information. Here, N is the number of all genes with a KEGG annotation, n is the number of DEGs (Differentially Expressed Genes) in N, M is the number of all genes annotated to specific pathways, and m is number of DEGs in M. Pathway enrichment analysis identifies significantly enriched metabolic pathways or signal transduction pathways associated with differentially expressed genes, comparing the whole genome background. KEGG terms with padj < 0.05 are significant enrichment.

A. Brassinosteroid

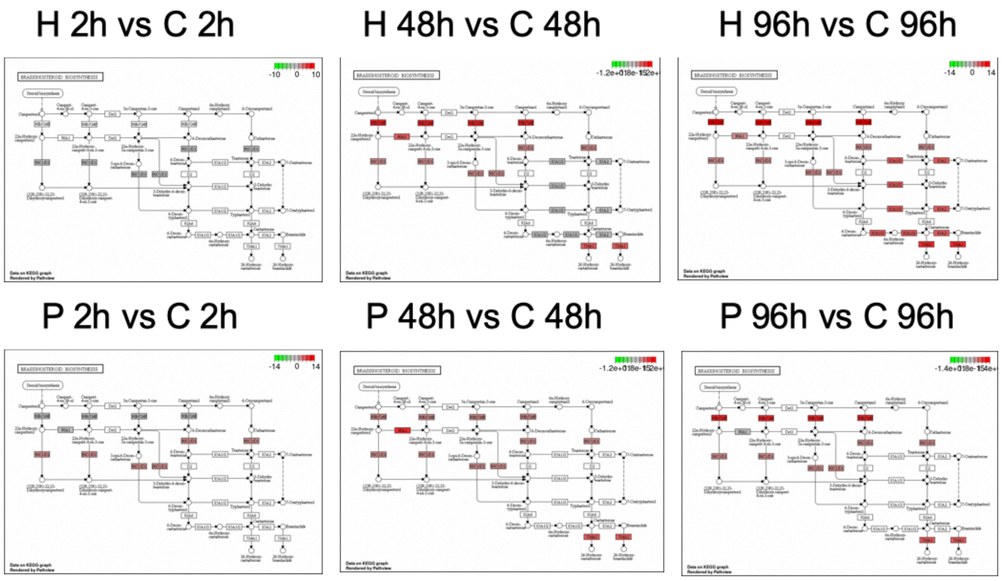


B. Zeatin

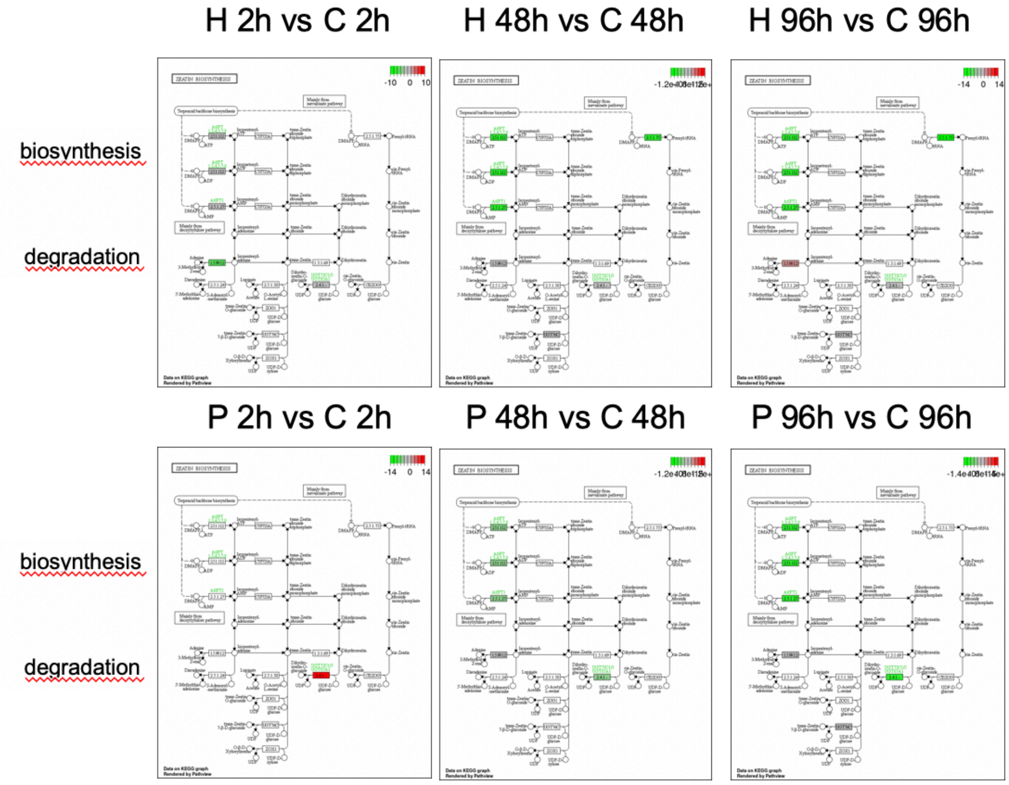

C. IAA

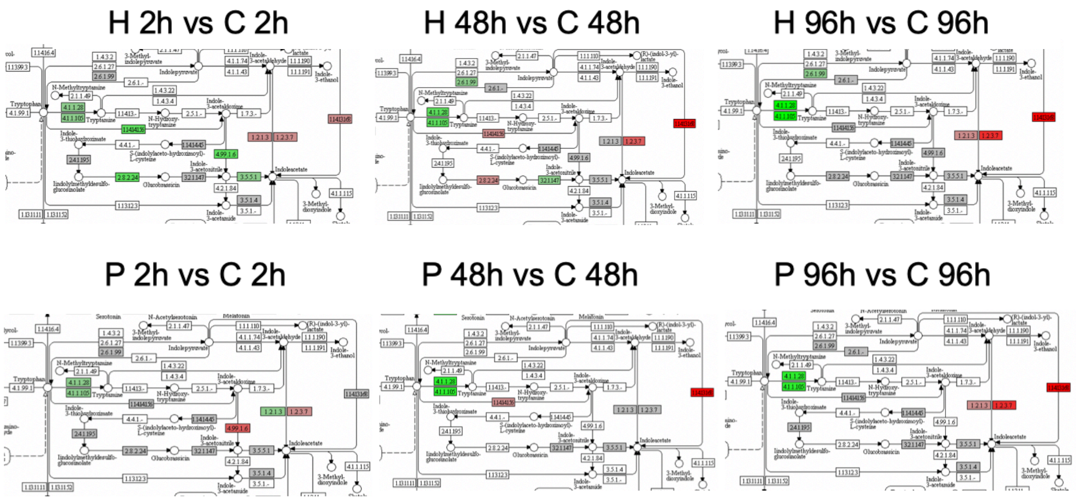


D. Gibberellin

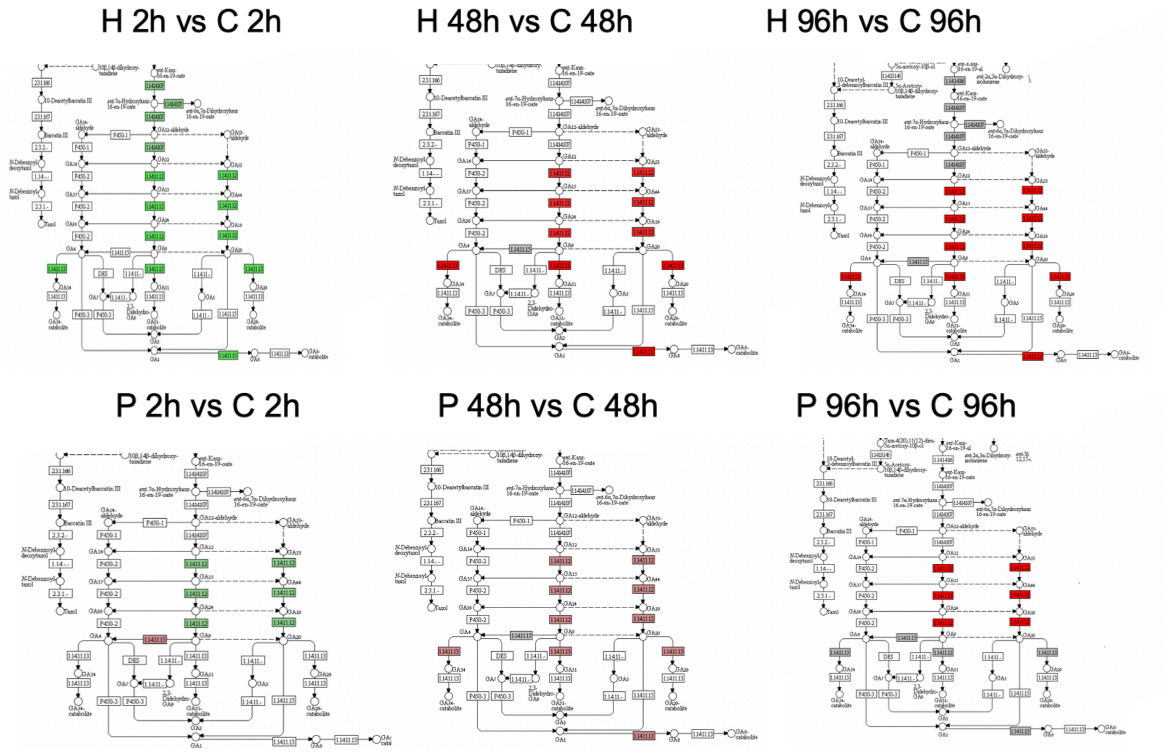

## E. ABA

H 2h vs C 2h

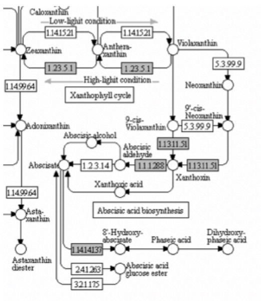

H 48h vs C 48h

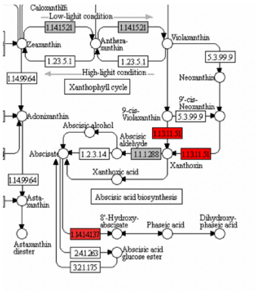

H 96h vs C 96h

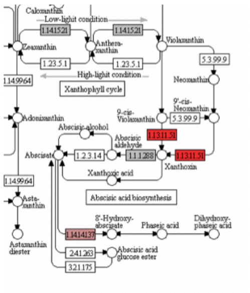

P 2h vs C 2h

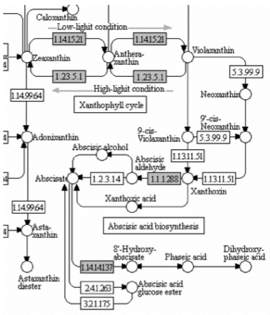

P 48h vs C 48h

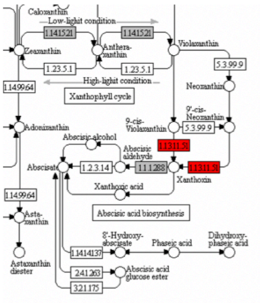

P 96h vs C 96h

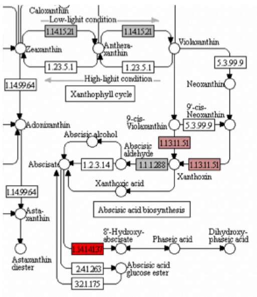

## F. Ethylene

H 2h vs C 2h

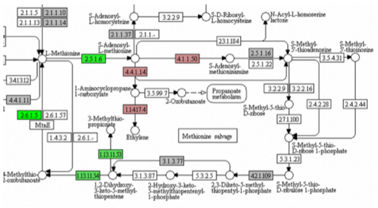

H 48h vs C 48h

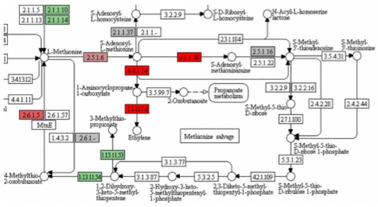

H 96h vs C 96h

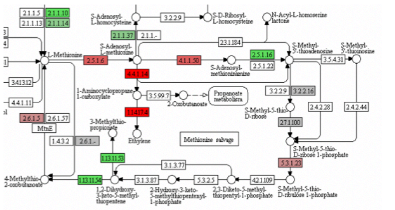

P 2h vs C 2h

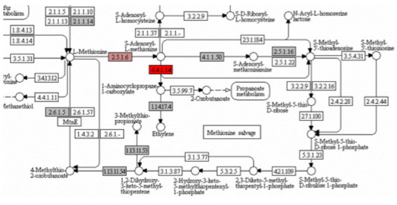

P 48h vs C 48h

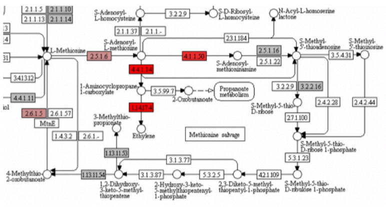

P 96h vs C 96h

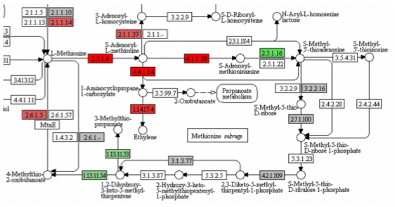

G. Jasmonate

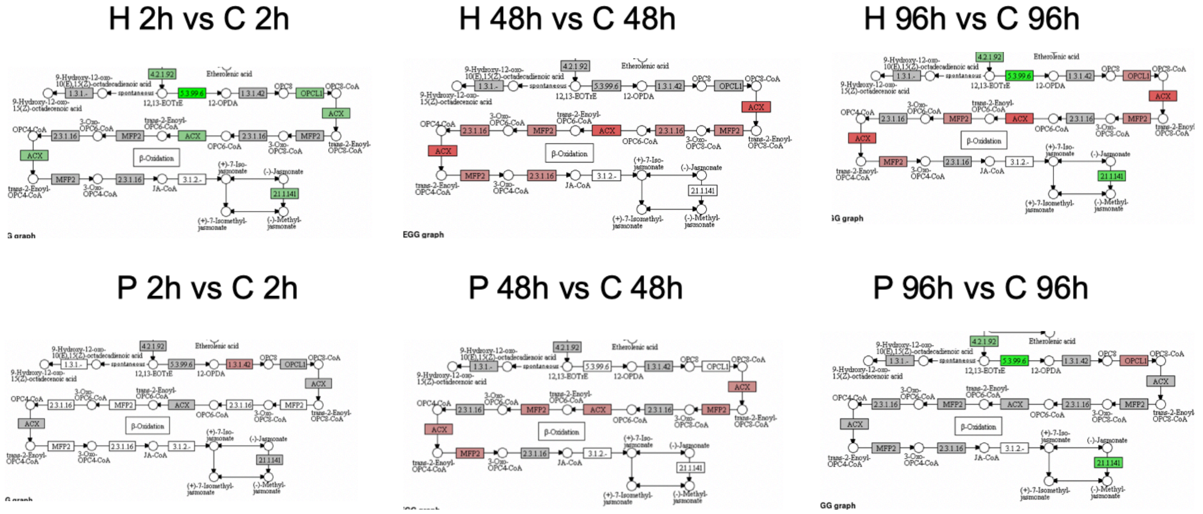


**Figure S6** Differentially regulated KEGG pathways for halauxifen (H) and picloram (P) treatments after 2, 48 and 96 hours compared to controls with respect to hormonal pathways. Green=downregulated; red=upregulated genes. (A) Brassinosteroid, (B) Zeatin (biosynthesis and degradation), (C) IAA, (D) Gibberellin, (E) ABA, (F) Ethylene, (G) Jasmonate.

## Supplementary tables

**Table S1.** Data quality summary as provided by Novogene. *

| Sample name | Raw reads | Clean reads | Raw bases | Clean bases | Error rate(%) | Q20(%) | Q30(%) | GC content(%) |
|---|---|---|---|---|---|---|---|---|
| c_2h_1 | 21770371 | 21158844 | 6.5G | 6.3G | 0.03 | 96.57 | 91.09 | 48.41 |
| c_2h_2 | 22438338 | 21776995 | 6.7G | 6.5G | 0.03 | 96.86 | 91.70 | 48.48 |
| c_2h_3 | 21495992 | 20838347 | 6.4G | 6.3G | 0.03 | 97.03 | 92.08 | 48.51 |
| p_2h_1 | 20630371 | 20065996 | 6.2G | 6.0G | 0.03 | 96.67 | 91.29 | 48.36 |
| p_2h_2 | 22492311 | 21982411 | 6.7G | 6.6G | 0.03 | 96.79 | 91.59 | 48.47 |
| p_2h_3 | 23296327 | 22667399 | 7.0G | 6.8G | 0.03 | 96.70 | 91.40 | 48.51 |
| h_2h_1 | 20485897 | 19890296 | 6.1G | 6.0G | 0.03 | 96.71 | 91.39 | 48.46 |
| h_2h_2 | 25411957 | 24585772 | 7.6G | 7.4G | 0.03 | 96.70 | 91.39 | 48.55 |
| h_2h_3 | 22416492 | 21059179 | 6.7G | 6.3G | 0.03 | 96.61 | 91.30 | 47.77 |
| c_48h_1 | 21546834 | 20987675 | 6.5G | 6.3G | 0.03 | 96.76 | 91.45 | 48.43 |
| c_48h_2 | 21783855 | 21191730 | 6.5G | 6.4G | 0.03 | 96.74 | 91.42 | 49.25 |
| c_48h_3 | 22843717 | 22184089 | 6.9G | 6.7G | 0.03 | 96.88 | 91.71 | 48.86 |
| p_48h_1 | 22336908 | 21707238 | 6.7G | 6.5G | 0.03 | 96.94 | 91.92 | 48.97 |
| p_48h_2 | 21165544 | 20505125 | 6.3G | 6.2G | 0.03 | 96.84 | 91.62 | 48.88 |
| p_48h_3 | 22792731 | 22090439 | 6.8G | 6.6G | 0.03 | 96.60 | 91.14 | 49.01 |
| h_48h_1 | 23102112 | 22498431 | 6.9G | 6.7G | 0.03 | 96.73 | 91.43 | 48.74 |
| h_48h_2 | 21093614 | 20589249 | 6.3G | 6.2G | 0.03 | 96.86 | 91.67 | 48.90 |
| h_48h_3 | 20486399 | 19916730 | 6.1G | 6.0G | 0.03 | 96.80 | 91.59 | 48.78 |
| c_96h_1 | 21493128 | 20800672 | 6.4G | 6.2G | 0.03 | 96.87 | 91.72 | 48.84 |
| c_96h_2 | 23955049 | 23158036 | 7.2G | 6.9G | 0.03 | 96.68 | 91.31 | 49.03 |
| c_96h_3 | 22924070 | 22073579 | 6.9G | 6.6G | 0.03 | 96.78 | 91.55 | 49.03 |
| p_96h_1 | 22235770 | 21521136 | 6.7G | 6.5G | 0.03 | 97.06 | 92.13 | 48.77 |
| p_96h_2 | 21228373 | 20627934 | 6.4G | 6.2G | 0.03 | 96.77 | 91.47 | 48.62 |
| p_96h_3 | 20086238 | 19465401 | 6.0G | 5.8G | 0.03 | 96.68 | 91.32 | 48.86 |
| h_96h_1 | 22982783 | 22298469 | 6.9G | 6.7G | 0.03 | 96.88 | 91.72 | 48.34 |
| h_96h_2 | 22882634 | 22225610 | 6.9G | 6.7G | 0.03 | 96.76 | 91.50 | 48.59 |
| h_96h_3 | 21079449 | 20466724 | 6.3G | 6.1G | 0.03 | 96.73 | 91.44 | 48.31 |

*Sample name: c= control, hal=halauxifen-methyl, pic=picloram 2,48,96 hrs after treatment, 1-3 replicates. Raw reads: reads count from the raw data, four rows as a unit, with statistics of reads count for every sequencing. Clean reads: Clean data are reads count filtered from raw data. Statistics method is similar with raw reads. All the following analysis is based on clean data. Raw bases: Base number of raw data. (number of raw reads) * (sequence length), converting unit to G. Clean bases: Base number of raw data after filtering. (number of clean reads) * (sequence length), converting unit to G. Error rate(%): base error rate of whole

sequencing. Q20(%): The percentage of the bases whose Q Phred values is greater than 20. (Number of bases with Q Phred value > 20) / (Number of total bases) *100. Q30(%): The percentage of the bases whose Q Phred values is greater than 30. (Number of bases with Q Phred value > 30) / (Number of total bases) *100. GC content(%): The percentage of G&C base numbers of total bases.(G&C base number) / (Total base number)*100.

**Table S2.** Mapping of reads. *

| Sample name | c_2h_1 | c_2h_2 | c_2h_3 | c_48h_1 | c_48h_2 | c_48h_3 | c_96h_1 | c_96h_2 | c_96h_3 |
|---|---|---|---|---|---|---|---|---|---|
| Total reads | 42317688 | 43553990 | 41676694 | 41975350 | 42383460 | 44368178 | 41601344 | 46316072 | 44147158 |
| Total mapped reads | 39005049 | 40264669 | 38611528 | 38699314 | 39322224 | 41146621 | 38518226 | 42733734 | 40801699 |
| Uniquely mapped reads | 36960217 | 38116634 | 36599766 | 36525326 | 37172940 | 38932536 | 36360260 | 40317626 | 38485990 |
| Multiple mapped reads | 2044832 | 2148035 | 2011762 | 2173988 | 2149284 | 2214085 | 2157966 | 2416108 | 2315709 |
| Total mapping rate | 92.17% | 92.45% | 92.65% | 92.20% | 92.78% | 92.74% | 92.59% | 92.27% | 92.42% |
| Uniquely mapping rate | 87.34% | 87.52% | 87.82% | 87.02% | 87.71% | 87.75% | 87.40% | 87.05% | 87.18% |
| Multiple mapping rate | 4.83% | 4.93% | 4.83% | 5.18% | 5.07% | 4.99% | 5.19% | 5.22% | 5.25% |

| Sample name | h_2h_1 | h_2h_2 | h_2h_3 | h_48h_1 | h_48h_2 | h_48h_3 | h_96h_1 | h_96h_2 | h_96h_3 |
|---|---|---|---|---|---|---|---|---|---|
| Total reads | 39780592 | 49171544 | 42118358 | 44996862 | 41178498 | 39833460 | 44596938 | 44451220 | 40933448 |
| Total mapped reads | 36822496 | 45519785 | 38790722 | 41347999 | 37987491 | 36640287 | 40932487 | 40726179 | 37494873 |
| Uniquely mapped reads | 34989046 | 43191291 | 36711620 | 39094325 | 35978179 | 34665143 | 38833733 | 38628893 | 35670925 |
| Multiple mapped reads | 1833450 | 2328494 | 2079102 | 2253674 | 2009312 | 1975144 | 2098754 | 2097286 | 1823948 |
| Total mapping rate | 92.56% | 92.57% | 92.10% | 91.89% | 92.25% | 91.98% | 91.78% | 91.62% | 91.60% |
| Uniquely mapping rate | 87.96% | 87.84% | 87.16% | 86.88% | 87.37% | 87.03% | 87.08% | 86.90% | 87.14% |
| Multiple mapping rate | 4.61% | 4.74% | 4.94% | 5.01% | 4.88% | 4.96% | 4.71% | 4.72% | 4.46% |

| Sample name | p_2h_1 | p_2h_2 | p_2h_3 | p_48h_1 | p_48h_2 | p_48h_3 | p_96h_1 | p_96h_2 | p_96h_3 |
|---|---|---|---|---|---|---|---|---|---|
| Total reads | 40131992 | 43964822 | 45334798 | 43414476 | 41010250 | 44180878 | 43042272 | 41255868 | 38930802 |
| Total mapped reads | 37095087 | 40659695 | 41909824 | 40179034 | 37954033 | 40757313 | 39934645 | 38234301 | 36005529 |
| Uniquely mapped reads | 35124072 | 38527852 | 39766529 | 38085572 | 35903375 | 38567866 | 37879953 | 36345181 | 34216685 |
| Multiple mapped reads | 1971015 | 2131843 | 2143295 | 2093462 | 2050658 | 2189447 | 2054692 | 1889120 | 1788844 |
| Total mapping rate | 92.43% | 92.48% | 92.45% | 92.55% | 92.55% | 92.25% | 92.78% | 92.68% | 92.49% |
| Uniquely mapping rate | 87.52% | 87.63% | 87.72% | 87.73% | 87.55% | 87.30% | 88.01% | 88.10% | 87.89% |
| Multiple mapping rate | 4.91% | 4.85% | 4.73% | 4.82% | 5.00% | 4.96% | 4.77% | 4.58% | 4.59% |

* Total reads: total clean reads used for analysis. Total mapped reads: numbers of reads being mapped on the genome, the ratio should higher than 70%. Uniquely mapped reads: numbers of reads being mapped on single position of the genome. Multiple mapped reads: numbers of reads being mapped on more than one position of the genome. Total mapping rate: (mapped reads)/(total reads)*100. Uniquely mapping rate: (uniquely mapped reads)/(total reads)*100. Multiple mapping rate: (multiple mapped reads)/(total reads)*100.

**Table S3.** GO is the abbreviation of Gene Ontology (http://www.geneontology.org/), which is a major bioinformatics classification system to unify the presentation of gene properties across all species. It includes three main branches: cellular component, molecular function and biological process. GO terms with padj < 0.05 are significant enrichment. The table shows a part of the complete analysis. *

| Category | GOID | Description | GeneRatio | BgRatio | pvalue | padj |
|---|---|---|---|---|---|---|
| BP | GO:0009733 | response to auxin | 123\|4509 | 398\|28297 | 5.01184403174344e-14 | 6.91634476380595e-11 |
| BP | GO:0015979 | photosynthesis | 92\|4509 | 323\|28297 | 7.56949568112022e-09 | 5.22295201997295e-06 |
| BP | GO:0042737 | drug catabolic process | 101\|4509 | 370\|28297 | 1.63418769451362e-08 | 7.11849342371176e-06 |
| BP | GO:0071555 | cell wall organization | 122\|4509 | 474\|28297 | 2.39561434158461e-08 | 7.11849342371176e-06 |

*Category: different class of GO id. CC, BP and MF are the abbreviation for Cellular_Component, Biological_Process and Molecular_Function respectively. GOID: unique identification id of Gene Ontology database. Description: function description of Gene Ontology. GeneRatio: ratio between the number of differentially expressed genes in each GO term and all differentially expressed genes that can be found in GO database. BgRatio: in background GO database, the ratio of all genes concerning this GO term to all genes. pvalue: statistics category term; abbreviation for probability value. padj: adjusted p-value. Generally, GO terms with Corrected_pValue < 0.05 are significant enrichment.

**Table S4**. Auxin-related GH3 genes and their expression as (A) ratio for the mean of three replicates. (B) For all genes the mean expression levels + SD are shown for controls and treatments in all upregulated combinations. No GH3.4 annotation since it is similar to GH3.2 (in *Arabidopsis thaliana* presumably a gene duplication). BrnaA: A genome; BrnaC: C genome. The small letters denote duplications of the respective genes as found in the annotation by Wang et al. (2019). GH3.5 of *A. thaliana* also catalyzes the conjugation of salicylic acid (SA) to amino acids. In yellow the only gene that was slightly downregulated is marked.

A.

| **Gene ID** | **Differential regulation** | | | | | | **Description (uniport)** |
|---|---|---|---|---|---|---|---|
| | **Halauxifen** | | | **Picloram** | | | |
| | **2** | **48** | **96** | **2** | **48** | **96 hrs** | |
| BnaA09R.GH3-1.a | 0 | 0 | 0 | 0 | 0 | 19 | Catalyzes the synthesis of indole-3-acetic acid (IAA)-amino acid conjugates. |
| BnaCX.GH3-1.a | 0 | 0 | 0 | 0 | 0 | 0 | |
| BnaA01.GH3-2.a | 12 | 3976 | 1457 | 27 | 421 | 226 | Catalyzes the synthesis of indole-3-acetic acid (IAA)-amino acid conjugates. Strongly reactive with Glu, Gln, Trp, Asp, Ala, Leu, Phe, Gly, Tyr, Met, Ile and Val. |
| BnaC01.GH3-2.a | 19 | 2565 | 2231 | 55 | 266 | 318 | |
| BnaA09.GH3-3.a | 11 | 3144 | 14574 | 28 | 974 | 3937 | Catalyzes the synthesis of indole-3-acetic acid (IAA)-amino acid conjugates. Strongly reactive with Glu, Gln, Trp, Asp, Ala, Leu, Phe, Gly, Tyr, Met, Ile and Val. |
| BnaC08.GH3-3.a | 31 | 1934 | 4783 | 119 | 590 | 2596 | |
| BnaA01.GH3-5.a | 0 | 19 | 65 | 0 | 0 | 14 | Catalyzes the synthesis of indole-3-acetic acid (IAA)-amino acid conjugates. Strongly reactive with Glu, Gln, Trp, Asp, Ala, Leu, Phe, Gly, Tyr, Met, Ile and Val. |
| BnaA03.GH3-5.b | 0 | 141 | 146 | 0 | 0 | 30 | |
| BnaC01.GH3-5.a | 0 | 17 | 22 | 0 | 0 | 0 | |
| BnaC07.GH3-5.b | 0 | 40 | 88 | 0 | 0 | 21 | |
| BnaA03.GH3-6.a | 3 | 7 | 11 | 0 | 4 | 8 | Catalyzes the synthesis of indole-3-acetic acid (IAA)-amino acid conjugates. Strongly reactive with Glu, Gln, Trp, Asp, |
| BnaAX.GH3-6.b | | | | | | | |

| | | | | | | | |
|---|---|---|---|---|---|---|---|
| | 0 | 8 | 9 | 4 | 0 | 0 | Ala, Leu, Phe, Gly, Tyr, Met, Ile and Val. Involved in auxin signal transduction. Inhibits shoot and hypocotyl cell elongation, and lateral root cell differentiation in light. |
| BnaC03.GH3-6.a | 0 | 11 | 125 | 0 | 5 | 89 | |
| BnaCX.GH3-6.b | 0 | 15 | 107 | 0 | 0 | 8 | |
| BnaA03.GH3-15.a | 0 | 2 | 0 | 0.78 | 2 | 3 | Indole-3-acetic acid-amido (IAA) synthetase that catalyzes the conjugation of amino acids to auxin specifically using the auxin precursor indole-3-butyric acid (IBA) and glutamine and, possibly, cysteine as substrates. Displays high catalytic activity with the auxinic phenoxyalkanoic acid herbicides 4-(2,4-dichlorophenoxy)butyric acid (2,4-DB) and to some extent 2,4-dichlorophenoxylacetic acid (2,4-D) as substrates, thus conferring resistance to herbicides. |
| BnaC03.GH3-15.a | 0 | 2 | 0 | 0 | 0 | 0 | |
| BnaA07.GH3-17.a | 0 | 31 | 21 | 0 | 0 | 0 | Catalyzes the synthesis of indole-3-acetic acid (IAA)-amino acid conjugates. Strongly reactive with Glu, Gln, Trp, Asp, Ala, Leu, Phe, Gly, Tyr, Met, Ile and Val. |
| BnaA08.GH3-17.b | 0 | 0 | 0 | 0 | 0 | 0 | |
| BnaA09.GH3-17.c | 0 | 33 | 40 | 0 | 0 | 0 | |
| BnaC08.GH3-17.a | 0 | 0 | 0 | 0 | 0 | 0 | |
| BnaCX.GH3-17.b | 0 | 24 | 48 | 0 | 0 | 0 | |
| BnaCX.GH3-17.c | 0 | 0 | 0 | 0 | 0 | 0 | |

B.

| Gene ID | Halauxifen 2 | | Control 2 | |
|---|---|---|---|---|
| | mean | SD | mean | SD |
| BnaA01.GH3-2.a | 73 | 10.5356538 | 6.33333333 | 3.05505046 |
| BnaC01.GH3-2.a | 64 | 29.0516781 | 3.33333333 | 3.21455025 |
| BnaA09.GH3-3.a | 41.3333333 | 19.5533458 | 3.66666667 | 0.57735027 |
| BnaC08.GH3-3.a | 41.6666667 | 13.5030861 | 1.33333333 | 0.57735027 |
| BnaA03.GH3-6.a | 49.3333333 | 14.571662 | 16 | 7 |
| | **Halauxifen 48** | | **Control 48** | |
| | mean | SD | mean | SD |
| BnaA01.GH3-2.a | 1325.333333 | 118.0056496 | 0.333333333 | 0.577350269 |
| BnaC01.GH3-2.a | 855 | 54.61684722 | 0.333333333 | 0.577350269 |
| BnaA09.GH3-3.a | 5239.333333 | 224.1346322 | 1.666666667 | 2.886751346 |
| BnaC08.GH3-3.a | 2578.666667 | 29.68725877 | 1.333333333 | 1.527525232 |
| BnaA01.GH3-5.a | 50 | 6 | 2.666666667 | 1.154700538 |
| BnaA03.GH3-5.b | 47 | 8.717797887 | 0.333333333 | 0.577350269 |
| BnaC01.GH3-5.a | 5.666666667 | 3.214550254 | 0.333333333 | 0.577350269 |
| BnaC07.GH3-5.b | 26.66666667 | 3.511884584 | 0.666666667 | 0.577350269 |
| BnaA03.GH3-6.a | 35.33333333 | 4.932882862 | 5.333333333 | 1.527525232 |
| BnaAX.GH3-6.b | 96.33333333 | 18.50225212 | 12.66666667 | 3.214550254 |
| BnaC03.GH3-6.a | 35 | 3 | 3.333333333 | 2.309401077 |
| BnaCX.GH3-6.b | 133.3333333 | 19.60442127 | 9 | 1 |
| BnaA03.GH3-15.a | 185 | 29.54657341 | 96 | 21 |
| BnaC03.GH3-15.a | 295.6666667 | 53.14445722 | 165 | 19.31320792 |
| BnaA07.GH3-17.a | 10.33333333 | 5.033222957 | 0.333333333 | 0.577350269 |
| BnaA09.GH3-17.c | 11 | 4.358898944 | 0.333333333 | 0.577350269 |
| BnaCX.GH3-17.b | 8 | 2.645751311 | 0.333333333 | 0.577350269 |
| | **Halauxifen 96** | | **Control 96** | |
| | mean | SD | mean | SD |
| BnaA01.GH3-2.a | 971 | 117.145209 | 0.666666667 | 1.154700538 |
| BnaC01.GH3-2.a | 743.6666667 | 98.39884823 | 0.333333333 | 0.577350269 |
| BnaA09.GH3-3.a | 4858 | 485.0268034 | 0.333333333 | 0.577350269 |
| BnaC08.GH3-3.a | 1594.333333 | 284.3876462 | 0.333333333 | 0.577350269 |
| BnaA01.GH3-5.a | 43 | 13.52774926 | 0.666666667 | 1.154700538 |
| BnaA03.GH3-5.b | 48.66666667 | 4.041451884 | 0.333333333 | 0.577350269 |
| BnaC01.GH3-5.a | 7.333333333 | 2.516611478 | 0.333333333 | 0.577350269 |

| | | | | |
|---|---|---|---|---|
| BnaC07.GH3-5.b | 29.33333333 | 14.571662 | 0.333333333 | 0.577350269 |
| BnaA03.GH3-6.a | 40.66666667 | 3.055050463 | 3.666666667 | 1.154700538 |
| BnaAX.GH3-6.b | 71.66666667 | 12.85820101 | 8 | 2.645751311 |
| BnaC03.GH3-6.a | 41.66666667 | 10.0166528 | 0.333333333 | 0.577350269 |
| BnaCX.GH3-6.b | 178.3333333 | 20.10804151 | 1.666666667 | 2.081665999 |
| BnaA07.GH3-17.a | 7 | 4 | 0.333333333 | 0.577350269 |
| BnaA09.GH3-17.c | 13.33333333 | 6.658328118 | 0.333333333 | 0.577350269 |
| BnaCX.GH3-17.b | 16 | 5 | 0.333333333 | 0.577350269 |
| | **Picloram 2** | | **Control 2** | |
| | mean | SD | mean | SD |
| BnaA01.GH3-2.a | 168.666667 | 39.1450295 | 6.33333333 | 3.05505046 |
| BnaC01.GH3-2.a | 182 | 19.5192213 | 3.33333333 | 3.21455025 |
| BnaA09.GH3-3.a | 103.666667 | 19.0087699 | 3.66666667 | 0.57735027 |
| BnaC08.GH3-3.a | 79.3333333 | 7.3711148 | 0.66666667 | 1.15470054 |
| BnaAX.GH3-6.b | 24.6666667 | 19.7568554 | 6.33333333 | 1.52752523 |
| | **Picloram 48** | | **Control 48** | |
| | mean | SD | mean | SD |
| BnaA01.GH3-2.a | 140.333333 | 11.2398102 | 0.33333333 | 0.57735027 |
| BnaC01.GH3-2.a | 88.6666667 | 14.4683563 | 0.33333333 | 0.57735027 |
| BnaA09.GH3-3.a | 1623.33333 | 134.183953 | 1.66666667 | 2.88675135 |
| BnaC08.GH3-3.a | 786.666667 | 77.3907833 | 1.33333333 | 1.52752523 |
| BnaA03.GH3-6.a | 20.6666667 | 7.63762616 | 5.33333333 | 1.52752523 |
| BnaC03.GH3-6.a | 16 | 3 | 3.33333333 | 2.30940108 |
| BnaA03.GH3-15.a | 215 | 47.0319041 | 96 | 21 |
| | **Picloram 96** | | **Control 96** | |
| | mean | SD | mean | SD |
| BnaA09R.GH3-1.a | 6.33333333 | 3.51188458 | 0.33333333 | 0.57735027 |
| BnaA01.GH3-2.a | 150.333333 | 16.2583312 | 0.66666667 | 1.15470054 |
| BnaC01.GH3-2.a | 106 | 13.114877 | 0.33333333 | 0.57735027 |
| BnaA09.GH3-3.a | 1312.33333 | 96.6247035 | 0.33333333 | 0.57735027 |
| BnaC08.GH3-3.a | 865.333333 | 96.9346859 | 0.33333333 | 0.57735027 |
| BnaA01.GH3-5.a | 9.33333333 | 2.081666 | 0.66666667 | 1.15470054 |
| BnaA03.GH3-5.b | 10 | 2.64575131 | 0.33333333 | 0.57735027 |
| BnaC07.GH3-5.b | 7 | 2.64575131 | 0.33333333 | 0.57735027 |
| BnaA03.GH3-6.a | 29 | 7 | 3.66666667 | 1.15470054 |
| BnaC03.GH3-6.a | 29.6666667 | 3.21455025 | 0.33333333 | 0.57735027 |

| | | | | |
|---|---|---|---|---|
| BnaCX.GH3-6.b | 14 | 5.56776436 | 1.66666667 | 2.081666 |
| BnaA03.GH3-15.a | 285.666667 | 32.3161466 | 108.666667 | 8.08290377 |
